# New Trichothecenes Isolated from the Marine Algicolous Fungus *Trichoderma* *brevicompactum*

**DOI:** 10.3390/md20020080

**Published:** 2022-01-18

**Authors:** Safwan Safwan, Shih-Wei Wang, George Hsiao, Sui-Wen Hsiao, Su-Jung Hsu, Tzong-Huei Lee, Ching-Kuo Lee

**Affiliations:** 1 Ph.D. Program in Clinical Drug Development of Herbal Medicine, College of Pharmacy, Taipei Medical University, Taipei 11031, Taiwan; safwan_afan@yahoo.com; 2 Department of Pharmacy, Faculty of Health Science, University of Muhammadiyah Mataram, Mataram 83127, Indonesia; 3 Department of Medicine, Mackay Medical College, New Taipei City 25245, Taiwan; shihwei@mmc.edu.tw; 4 Graduate Institute of Natural Products, College of Pharmacy, Kaohsiung Medical University, Kaohsiung 80708, Taiwan; 5 Graduate Institute of Medical Sciences, College of Medicine, Taipei Medical University, Taipei 11031, Taiwan; geohsiao@tmu.edu.tw; 6 Department of Pharmacology, School of Medicine, Taipei Medical University, Taipei 11031, Taiwan; 7 Ph.D. Program in Drug Discovery and Development Industry, College of Pharmacy, Taipei Medical University, Taipei 11031, Taiwan; d343106004@tmu.edu.tw; 8 School of Pharmacy, College of Pharmacy, Taipei Medical University, Taipei 11031, Taiwan; juliashu1101@gmail.com; 9 Institute of Fisheries Science, National Taiwan University, Taipei 10617, Taiwan

**Keywords:** *Trichoderma brevicompactum*, trichothecenes, marine alga, endophytic fungus, cytotoxic activities

## Abstract

Eight trichothecenes, including four new compounds **1**–**4** and four known entities **5**–**8**, together with one known cyclonerane (**9**) were isolated from the solid-state fermentation of *Trichoderma* *brevicompactum* NTU439 isolated from the marine alga *Mastophora rosea*. The structures of **1**–**9** were determined by 1D/2D NMR (nuclear magnetic resonance), MS (mass spectrometry), and IR (infrared spectroscopy) spectroscopic data. All of the compounds were evaluated for cytotoxic activity against HCT-116, PC-3, and SK-Hep-1 cancer cells by the SRB assay, and compound **8** showed promising cytotoxic activity against all three cancer cell lines with the IC_50_ values of 3.3 ± 0.3, 5.3 ± 0.3, and 1.8 ± 0.8 μM, respectively. Compounds **1**–**2**, **4**–**6**, and **7**–**8** potently inhibited LPS-induced NO production, and compounds **5** and **8** showed markedly inhibited gelatinolysis of MMP-9 in S1 protein-stimulated THP-1 monocytes.

## 1. Introduction

Fungi are a potential source of drug leads that researchers are still seeking [1,2]. The discovery of new compounds with unique structural diversity and low molecular weight presents opportunities for discovering bioactive natural products from fungi [2]. Although less explored, marine fungi are an important and rich source for the discovery of new compounds. A number of new compounds from marine fungi have been discovered from various sources, including extreme sea environment. These compounds showed various activities including anticancer, antimicroalgal, antibacterial, and antiviral effects [3,4,5]. The vast symbiotic relationships and diversity of many marine organisms has caused marine fungi to distribute in almost all marine habitats, including from marine ray-finned fish, sponges, mangroves, and algae [3,5,6,7]. Among them, marine algae-derived fungi offer opportunities and attract attention because they produce secondary metabolites with unique chemical diversity and various pharmacological properties [4,6,8,9,10,11].

*Trichoderma* species are commonly present in all soils and various habitats, including marine habitats and marine sediments [4,8,12,13]. The genus *Trichoderma* produces metabolites with trichothecene scaffold and other skeletal metabolites including epipolythiodioxopiperazines, peptides, pyrones, butenolides, pyridones, anthraquinones, and steroids, along with various low molecular weight compounds [14]. Trichothecenes are a family of sesquiterpenes with a tetracyclic 12,13-epoxytrichothec-9-ene ring that have been identified in several fungal genera including *Trichoderma*, *Myrothecium*, *Trichothecium*, and *Fusarium* [4,7,15,16]. Trichothecene derivatives including trichodermin, HT-2 toxin, deoxynivalenol, and satratoxin were reported to exhibit activities such as cytotoxic, antiphytopathogenic, antimicroalgal, antifungal, antimalarial, antiviral, antibiotic, and antileukemic effects [4,12,15,16,17,18,19]. In this study, a chemical investigation was performed on solid fermented products of *T. brevicompactum* NTU439, which has resulted in the isolation and identification of four new trichothecenes **1**–**4**, together with five known compounds. Characterization of the new compounds and bioactivities of all compounds are described herein.

## 2. Results

### 2.1. Chemical Characterization of the Produced Compound

**Figure 1:**
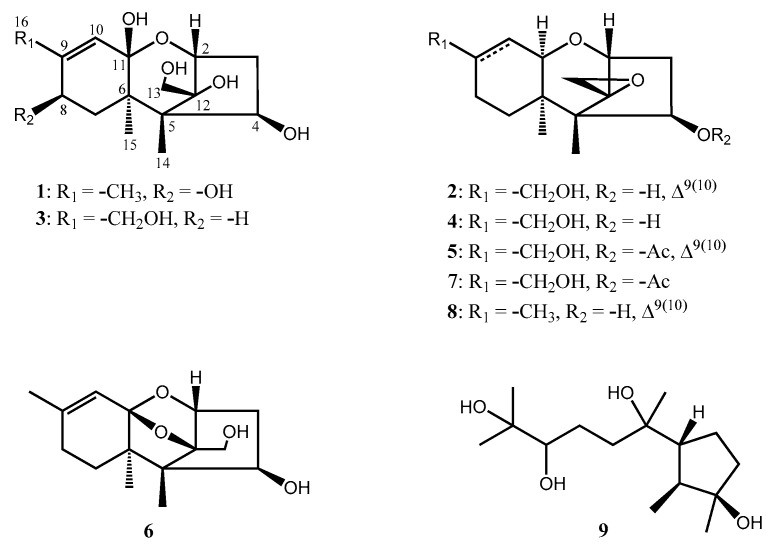
Structures of compounds **1**–**9**.

In this study, in an attempt to identify bioactive compounds from ethyl acetate extract of the solid culture of *T. brevicompactum* NTU439 isolated from a marine alga *M. rosea*, we isolated four new trichothecenes (**1**–**4**), together with five known compounds, trichoderminol (**5**) [17], trichodermarin A (**6**) [12], trichodermarin E (**7**) [12], trichodermol (**8**) [20], and cycloneran-3,7,10,11-tetraol (**9**) [21] (Figure 1), and identified them by their spectral data and the comparison of spectroscopic data with the literature.

**Figure 2:**
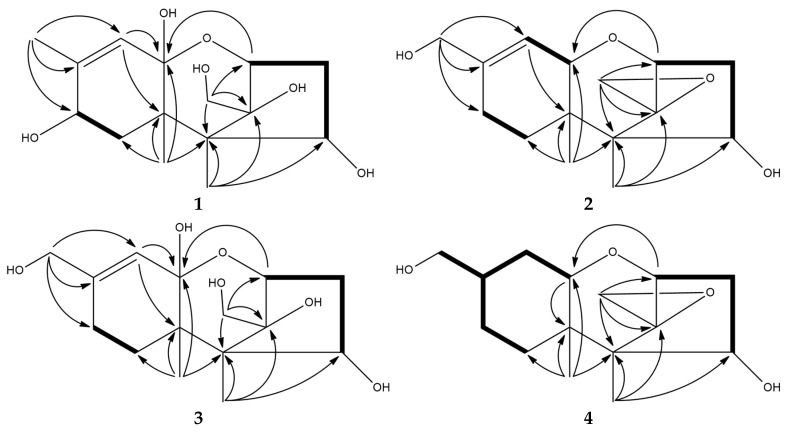
Key HMBC (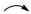) and COSY (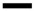) correlations of compounds **1**–**4**.

Compound **1** was obtained in the form of a colorless oil with the molecular formula C_15_H_24_O_6_ deduced from a pseudo-molecular ion [M − H_2_O + H]^+^ at *m/z* 283.1536 (calcd. 283.1540) in the HRESIMS supported by ^13^C NMR spectrum. The IR spectrum revealed a hydroxy group and a double bond at 3365 and 1647 cm^–1^, respectively. The ^1^H NMR spectrum showed a signal at *δ*_H_ 5.39 (q, *J* = 1.2 Hz, 1H), attributable for one olefinic proton, three oxymethine protons (*δ*_H_ 4.15, d, *J* = 5.3 Hz, 1H; *δ*_H_ 4.04, dd, *J* = 7.5, 1.6 Hz, 1H; *δ*_H_ 4.01, d, *J* = 5.6 Hz, 1H), two oxygenated methylene protons (*δ*_H_ 3.85 and 3.83, d, *J* = 12.0 Hz, each 1H), two sets of nonequivalent methylene protons (*δ*_H_ 2.47, dd, *J* = 16.3, 7.5 Hz, 1H; *δ*_H_ 1.77, ddd, *J* = 16.3, 5.3, 1.6 Hz, 1H; *δ*_H_ 2.05, dd, *J* = 14.2, 5.6 Hz, 1H; *δ*_H_ 1.67, dd, *J* = 14.2, 1.3 Hz, 1H), and three methyl groups (*δ*_H_ 1.87, d, *J* = 1.2 Hz, 3H; *δ*_H_ 1.04, s, 3H; *δ*_H_ 0.99, s, 3H) (Table 1, Figure S1, Supplementary Material). The ^13^C NMR, in combination with DEPT and HSQC spectra, indicated the presence of two olefinic carbons signals (*δ*_C_ 118.2 and 144.7), one dioxygenated carbons (*δ*_C_ 107.5), three oxymethine carbons (*δ*_C_ 81.2, 74.6, and 66.7), two quaternary carbons (*δ*_C_ 54.2 and 46.9), one oxygenated carbon (*δ*_C_ 95.0), two methylene carbons (*δ*_C_ 40.9 and 38.9), one oxymethylene carbon (*δ*_C_ 58.1), and three methyl carbons (*δ*_C_ 19.1, 16.0, and 9.6) (Table 2). Key COSY cross-peaks corroborated two fragments of H-2 (*δ*_H_ 4.15)/H_2_-3 (*δ*_H_ 2.47 and 1.77)/H-4 (*δ*_H_ 4.04) and H_2_-7 (*δ*_H_ 2.05 and 1.67)/H-8 (*δ*_H_ 4.01) (Figure 2). Key correlations from HMBC spectrum were H-2 (*δ*_H_ 4.15)/C-11 (*δ*_C_ 107.5), C-12 (*δ*_C_ 95.0), C-5 (*δ*_C_ 54.2), and C-13 (*δ*_C_ 58.1); H_2_-13 (*δ*_H_ 3.85 and 3.83)/C-12 (*δ*_C_ 95.0), C-5 (*δ*_C_ 54.2) and C-2 (*δ*_C_ 81.2); H_3_-14 (*δ*_H_ 0.99)/C-5 (*δ*_C_ 54.2), C-6 (*δ*_C_ 46.9), C-12 (*δ*_C_ 95.0), and C-4 (*δ*_C_ 74.6); H_3_-15 (*δ*_H_ 1.04)/C-5 (*δ*_C_ 54.2), C-6 (*δ*_C_ 46.9), C-7 (*δ*_C_ 38.9), and C-11 (*δ*_C_ 107.5); and H_3_-16 (*δ*_H_ 1.87)/C-9 (*δ*_C_ 144.7), C-8 (*δ*_C_ 66.7), and C-10 (*δ*_C_ 118.2) (Figure 2). The NOESY correlations of H-8/H_3_-15, H-4/H_3_-15, and H_2_-13/H-2 and H_3_-14 indicated that H-4, H-8, and H_3_-15 and H_2_-13, H_3_-14, and H-2 were on the same side of the ring system in **1**. From the molecular modelling (ChemBio3D Ultra 12.0) of compound **1** at minimized energy state, the predicted distances between H-10 and H_2_-13 of **1** with β-oriented OH-11 or α-oriented OH-11 were 5.1 or 2.9 Å, respectively. In general, the NOESY correlation signal between two protons can be found only when their distance was lower than 5 Å. In the NOESY spectrum of compound **1,** no crosspeak of H-10/H_2_-13 was not observed. Thus, the stereochemistry of OH-11 was determined to be β-oriented, as shown in Figure 1.

Compound **2** was obtained as colorless oil, and its molecular formula was determined to be C_15_H_22_O_4_ deduced from HRESIMS deduced from a molecular ion [M + H]^+^ at *m/z* 267.1588 (calcd. 267.1590). The IR spectrum indicated a hydroxy group and a double bond with absorption bands at 3396 and 1652 cm^–1^, respectively. The resonances on ^1^H (Table 1) and ^13^C (Table 2) supported by DEPT spectroscopic data of compound **2** were consistent with those of compounds **5** and **8**, except for some differences, including the acetoxy group signal in **5** replaced by a hydroxy in **2** and the methyl group of compound **8** substituted by an oxygenated methylene in **2** [*δ*_H_ 3.97 (br, 1H, H-16a) and 3.94 (br, 1H, H-16b); *δ*_C_ 64.8 (C-16)]. Key HMBC (Figure 2) correlations of H-16 (*δ*_H_ 3.97 and 3.94, 2H)/C-8 (*δ*_C_ 23.0), C-9 (*δ*_C_ 142.9), and C-10 (*δ*_C_ 118.1) indicated the oxygenated methylene functionality was located at the C-9 (*δ*_C_ 142.9) in **2**. Thus, the structure of **2** was assigned to be as shown.

Compound **3** was obtained as a colorless gum with elemental formula of C_15_H_24_O_6_ deduced by HRESIMS [M − H_2_O + H]^+^ at *m*/*z* 283.1537 (calcd. 283.1540). The ^1^H (Table 1) and ^13^C NMR (Table 2) supported by DEPT spectroscopic data of compound **3** were consistent with compound **1**, except for the absence of a methyl group (*δ*_H_ 1.87, d, *J* = 1.2 Hz, 3H; *δ*_C_ 19.1) and an oxymethine (*δ*_H_ 4.01, d, *J* = 5.6 Hz, 1H; *δ*_C_ 66.7) in compound **1**, which were substituted by an oxygenated methylene (*δ*_H_ 3.98, br, 2H; δ_C_ 65.6) and a methylene (*δ*_H_ 2.18 and 2.05, m, 2H; *δ*_C_ 25.3) in compound **3**, respectively. Key HMBC correlations of H-16 (*δ*_H_ 3.98, br s, 2H) to C-9 (*δ*_C_ 149.5), C-8 (*δ*_C_ 25.3), and C-10 (*δ*_C_ 116.7) confirmed the position of oxygenated methylene and methylene to be at C-16 and C-8, respectively, in compound **3** (Figure 2). The structure of compound **3** was thus determined to be as shown.

Compound **4** was obtained as a colorless gum with elemental formula of C_15_H_24_O_4_ determined by ^13^C NMR and HRESIMS [M + H]^+^ at *m/z* 269.1743 (calcd. 269.1747) analysis. Combination of IR, ^1^H (Table 1), ^13^C NMR (Table 2), and HMBC (Figure 2) spectrum data confirmed that compound **4** was almost similar to compound **7**, indicating that **4** possessed an identical skeleton to that of **7** except for acetoxy group signal in **7** replaced by a hydroxy in C-2; these results, together with the appearance of the oxymethine carbons (C-4, *δ*_C_ 72.3) in **4**, were much more upfield than that in **7**. The COSY spectrum data supported these results, in that the correlations of H-9 (*δ*_H_ 1.75) to H_ab_-16 (*δ*_H_ 3.74 and 3.47), H_ab_-10 (*δ*_H_ 1.92 and 1.59), and H_ab_-8 (*δ*_H_ 1.70 and 1.64); Hab-8 to H_ab_-7 (*δ*_H_ 1.98 and 1.17); H_ab_-10 to H-11 (3.39). The NOESY correlation peaks of compound **4** were similar to those of compound **7**, which is also a tricothecene-based compound [12].

### 2.2. Functional Characterization of the Produced Compounds

**Figure 3:**
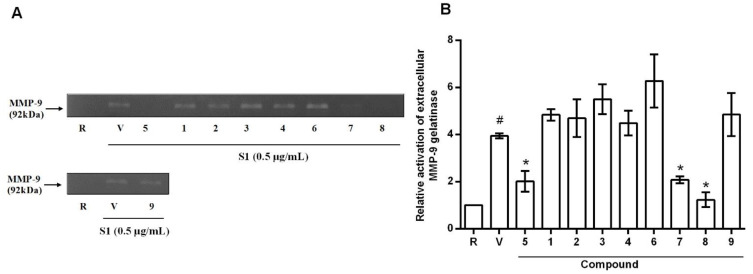
Gelatinolytic activity of MMP-9 in S1 protein-stimulated THP-1 monocytes. Zymogram shows the different activity of MMP-9 upon treatment of THP-1 monocytes with the different compounds (**A**). The relative quantification of gelatinase MMP-9 is reported in (**B**). R: resting (condition with no stimulation); V: vehicle (DMSO) with S1 protein THP-1 monocytes cells. Data represent means ± S.D. # *p* < 0.01 compared with the resting; * *p* < 0.05 compared with the vehicle.

All nine compounds were tested for cytotoxic activity against three cancer cell lines (colorectal cancer cells (HCT-116), prostate cancer cells (PC-3), and hepatocellular carcinoma cells (SK-Hep-1)) by SRB assay, NO production inhibitory activity in LPS-activated microglial BV-2 cell, and gelatinolysis of extracellular of MMP-9 in human THP-1 monocytic cells S1 protein-simulated. Trichodermin, known for its anticancer activities, was used as the positive control. The results (Table 3) revealed that trichodermol (compound **8**) showed potent cytotoxic activity against the three cancer cell lines, particularly against prostate cancer cells (PC-3), with the IC_50_ values of 3.3 ± 0.3, 5.3 ± 0.3, and 1.8 ± 0.8 μM, respectively. Trichoderminol (compound **5**) showed moderately cytotoxic activity against the three cancer cell lines, particularly in prostate cancer cells, and trichodermarin E (compound **7**) showed weak cytotoxic activity against the three cancer cell lines (see Table 3). The new compounds (**1**, **2**, and **4**) and the known compounds (**5**–**8**) potently inhibited LPS-induced NO production in BV-2 cells already at a concentration of 10 μM. Compounds **5** and **8** were toxic to BV-2 cells, with survival of cells at a concentration 10 µM of 62.9 ± 3.7% and 63.3 ± 6.4%, respectively. Compounds **4**, **6**, and **9** showed weak inhibitory activities with no toxic effect against BV-2 cells at a concentration of 10 μM. Furthermore, compound **7** displayed a strong inhibitory activity of LPS-induced NO production with IC_50_ value of 5.2 ± 0.4 μM (curcumin was used for comparison of bioactivity (IC_50_ = 2.7 ± 0.4 μM)). Compounds **5**, **7**, and **8** exerted attenuation of S1 protein-stimulated MMP-9-mediated gelatinolysis of 66.1 ± 3.1%, 63.4 ± 0.4%, and 92.1 ± 1.9% at 10 µM, respectively (Figure 3). Trichodermol (**8**) is a member of a family of fungal metabolites that show broad-spectrum antifungal activities and moderate cytotoxic activity against the MCF-7 line (breast carcinoma) [20,22]. Trichoderminol (compound **5**) was first isolated in 2017 and has antifungal, antimicroalgal, and antiviral activities [4,17].

## 3. Discussion

Trichothecenes comprise a group of sesquiterpenes that have been reported both from fungal cultures such as those of *Myrothecium* spp., *Trichothecium* spp., and *Fusarium* spp., as well as from some higher plants such as *Bacchairis coridifolia*, *B. artemisioides*, *Ficus fistulosa*, and *Rhaphidophora decursiva* [7,15,16,18,23,24]. Among these, some of the trichothecene-producing fungal species were marine-derived, such as *Myrothecium* sp. and *Trichoderma* sp. [7,12]. Structurally, trichothecenes are a family of sesquiterpenoids composed of a tricyclic 12,13-epoxytrichothec-9-ene (trichothecene) ring. On the basis of substitutions on the tricyclic moiety, trichothecenes are subcategorized into four types (A, B, C, and D), and over 200 compounds have been isolated [25]. Type A is the simplest structure, being non-substituted, hydroxylated, or esterified. All compounds that we have isolated in this report can be categorized as type A. The structure–cytotoxic activity relationship of trichothecenes has been extensively researched previously [2,25]. In particular, trichothecene with the C-12,13-epoxy ring, the double bond between C-9 and C-10 in A ring, and OH-4 have been identified as key structural features contributing to their toxicity [26,27,28]. In this study, we observed that in compounds **1**, **3**, and **6**, the C-12,13-epoxy ring is hydrolyzed to be opened as well as the double bond between C-9 and C-10 in A ring that led to no cytotoxic activity against three cancer cell lines. On the contrary, compounds **4** and **7** with the C-12,13-epoxy ring, without a double bond between C-9 and C-10 in A ring, showed reduced cytotoxicity, but the presence of -OAc instead of -OH at C-4 could increase cytotoxic activity [28]. On the other hand, the new compounds **2** and **4** and the know compounds **5**, **7**, and **8** potently inhibited LPS-induced NO production in BV-2 cells. Further, the presence of a double bond between C-9 and C-10, and OH-4 could also increase activity in the inhibition of MMP-9 gelatinolysis, especially in compound **8**. These findings provide evidence that compounds **5**, **7**, and **8** may serve as potential drugs for neuroinflammation-related diseases and for anticancer treatment.

## 4. Materials and Methods

### 4.1. General Experimental Procedures

Optical rotations data were measured on a JASCO P-2000 polarimeter (Tokyo, Japan). 1D and 2D NMR spectrum data were recorded on Agilent DD2 600 MHz spectrometer (Agilent Technologies, Santa Clara, CA, USA). High-resolution ionization mass spectra were acquired on a Q Exactive Plus Hybrid Quadrupole-Orbitrap Mass Spectrometer (Thermo Fisher Scientific, Bremen, Germany). Infrared (IR) spectra data were recorded on a JASCO FT/IR 4100 spectrometer (Tokyo, Japan). Open column chromatography was using Sephadex LH-20 (GE Healthcare, Uppsala, Sweden), and thin-layer chromatography was performed using silica gel 60 F_254_ plates (0.2 mm) (Merck, Darmstadt, Germany). An HPLC pump L-7100 (Hitachi, Naka, Japan) equipped with a refractive index detector (Bischoff, Leonberg, Germany) was used for compound purification. All the organic solvents were purchased from Merck (Darmstadt, Germany).

### 4.2. Strain Isolation and Fermentation

*T. brevicompactum* NTU439 was isolated from *M. rosea* marine alga, which was collected from Yilan coast (24°57′13.9″ N 121°54′49.3″ E), Taiwan, in June 2016, and was identified on the basis of sequencing of the internal transcribed spacer (ITS) regions of the rDNA. The sequence of fungus NTU439 matched as *T. brevicompactum* by A BLAST search sequence (GenBank accession no. OK217197). The mycelium of *T. brevicompactum* NTU439 was inoculated into 250 mL flasks, each containing 50 g of brown rice (Santacruz, Taipei, Taiwan) and 15 mL deionized water with 1% KH_2_PO_4_, 1% sodium tartrate, and 2% yeast extract (Becton, Dickinson and Company, Sparks, MD, USA). The fermentation process was conducted under aeration for 30 days at 27–30 °C.

### 4.3. Extraction and Purification of Secondary Metabolites

Solid state fermentation products were lophilized, ground into powder, and then extracted twice with methanol (equal volume) and concentrated to obtain the crude extract (4.4 g). The crude extracts were suspended in an equal volume of deionized H_2_O and partitioned three times with equal volumes of ethyl acetate, *n*-hexane, and *n*-butanol, separately. The ethyl acetate layer was concentrated to obtain dried extract (2.3 g), re-dissolved in 20 mL methanol, and then subjected to Sephadex LH-20 CC (3.0 i.d. × 67.0 cm) using methanol as the eluent (2.1 mL/min) to give 36 fractions (21.0 mL/fr). The fractions were combined into 10 pools (Fr.A–I) on the basis of the results of TLC analysis. The Fr.D was further purified on a semi-preparative column (Phenomenex Luna PFP, 5 μm, 10 × 250 mm, Torrance, CA, USA) using 50% methanol containing 0.1% formic acid as eluent with a flow rate of 2 mL/min to give **5** (*t*_R_: 12.75 min, 5.43 mg) and six fraction (Fr.D1-D6). Further purification of Fr.D1 on a semi-preparative column (Thermo Hypersil HS C18, 5 μm, 10 × 250 mm, Bellefonte, PA, USA) eluted by 40% methanol (2 mL/min) gave **1** (*t*_R_: 7.51 min, 4.83 mg) and Fr.D1-2, and then the Fr.D1-2 was re-chromatographed on a RP-HPLC column (Phenomenex Luna PFP, 5 μm, 10 × 250 mm, Torrance, CA, USA) using 30% methanol (2 mL/min) as mobile phase to give **2** (*t*_R_: 33.42 min, 4.54 mg) and **3** (*t*_R_: 35.23 min, 4.48 mg). Further purification of Fr.D3, Fr.D5, and Fr.D6 was performed on a semi-preparative column (Thermo Hypersil HS C18, 5 μm, 10 × 250 mm, Bellefonte, PA, USA) eluted by methanol (40%, 35%, and 30%, respectively) at a flow rate of 2 mL/min to give **4** (*t*_R_: 12.12 min, 4.75 mg) from Fr.D3, **6** (*t*_R_: 16.3 min, 7.09 mg) from Fr.D5, and **7** (*t*_R_: 17.01 min, 10.55 mg) and **8** (*t*_R_: 20.21 min, 4.03 mg) from Fr.D6. Compound **9** (*t*_R_: 13.21 min, 4.45 mg) was obtained from Fr.C purified on a semi-preparative column (Phenomenex Luna PFP, 5 μm, 10 × 250 mm, Torrance, CA, USA) using 55% methanol containing 0.1% formic acid as eluent at a flow rate of 2 mL/min.

### 4.4. Cell Culture

The colorectal cancer cell line HCT-116, prostate cancer cell line PC-3, and hepatocellular carcinoma cell line SK-Hep-1 were purchased from the American Type Cell Culture Collection (Manassas, VA, USA). Cell culture was performed following the procedure of our previous reports [6]. In summary, the cells were maintained in DMEM medium containing fetal bovine serum (FBS), penicillin, and streptomycin in humidified air containing 5% CO_2_ at 37 °C.

### 4.5. Biologic Assay for Cytotoxic Activity

The SRB assay was used to determine the cytotoxic activity according to previously described procedures [6]. The HCT-116, PC-3, and SK-Hep-1 cancer cells were seeded onto 96-well plates in a density of 5 × 10^3^ cells per well. Overnight, cells were treated with the tested compounds for 48 h.

### 4.6. Biologic Assay for Relative Gelatinolysis by MMP-9

The relative gelatinolysis of by in human THP-1 monocytic cells MMP-9 was performed following the procedure of our previous reports [29]. Briefly, the THP-1 cells were subcultured and developed for 24 h in 24-well plates using serum-free medium. After the cell’s adhesion and growth, they were treated with 10 µM of compounds or vehicle (DMSO) followed by S1 protein (0.5 µg/mL) stimulation for 24 h before analysis of the MMP-9 gelatinolysis. The medium was collected and mixed with a non-reducing buffer that contains Tris–HCl, glycerol, SDS, and bromophenol blue (500 mM, 25%, 10%, and 0.32%, respectively), pH 6.8, and electrophoresed on gels containing 1 mg/mL of gelatin. The gels were washed with 2% Triton X-100 after electrophoresis and then incubated with reacting buffer containing Tris–base, NaCl, CaCl_2_, and Brij 35, pH 7.5, for 17 h at 37 °C. After incubation, the gels were fixed with 7% acetic acid and 40% methanol (*v*/*v*) for 30 min and then stained with Colloidal Brilliant Blue G in 25% methanol for 40 min. Clear zones (bands) against the blue background indicated the presence of gelatinolysis by MMP-9. Gelatinolytic zones were imaged and analyzed. The viability of THP-1 monocytic cells was measured using MTT assay after incubation with compounds or vehicle (DMSO) for 24 h.

### 4.7. Biologic Assay for Anti-Neuroinflammatory Activity

Culturing procedure and media composition for culturing of the mouse microglial BV-2 cell line was performed as described in our previous report [30]. The cells were pretreated with a concentration of compounds or vehicle (DMSO) for 15 min and then stimulated with LPS for 24 h. Cellular viability of BV-2 cells treated for the 24 h with compounds was measured by a colorimetric assay of MTT reduction [31]. The levels of nitrite were measured at 550 nm using a microplate reader (MRX) for evaluation of nitric oxide production as we have previously described [32]. Sodium nitrite was used as a standard, and curcumin was used as the positive control.

## 5. Conclusions

In this report, eight trichothecenes, including four new trichothecenes **1**–**4** and four known compounds **5**–**8**, along with one known cyclonerane, **9**, were isolated from the marine algae *M. rosea*-derived fungus *T. brecicompactum* NTU439. Functional characterization of all the isolates was evaluated by cytotoxic activity against three cancer cell lines (HCT-116, PC-3, and SK-Hep-1), inhibition of LPS-induced NO production, and inhibition of gelatinolysis by MMP-9. Of the compounds identified, trichoderminol (**5**), trichodermarin E (**7**), and trichodermol (**8**) exhibited promising cytotoxicity against three cancer cell lines and inhibition of both MMP-9 gelatinolysis and LPS-induced NO production.

## Figures and Tables

**Table 1:** ^1^H NMR data (600 MHz, MeOH-*d*_4_) of compounds **1**–**4**.

Position	1	2	3	4
	*δ*_H_ (*J* in Hz)	*δ*_H_ (*J* in Hz)	*δ*_H_ (*J* in Hz)	*δ*_H_ (*J* in Hz)
2	4.15, d (5.3)	3.67, d (5.3)	4.18, d (5.3)	3.66, d (5.3)
3a	2.47, dd (16.3, 7.5)	2.49, dd (15.1, 7.6)	2.50, dd, (16.4, 7.6)	2.37, dd (15.0, 7.8)
3b	1.77, ddd (16.3, 5.3, 1.6)	1.87, ddd (15.1, 5.3, 3.2)	1.80, ddd, (16.4, 5.3, 1.8)	1.80, ddd (15.0, 5.3, 3.5)
4	4.04, dd (7.5, 1.6)	4.41, dd (7.6, 3.5)	4.05, dd (7.6, 1.5)	4.29, dd (7.8, 3.5)
7a	2.05, dd (14.2, 5.6)	1.91, dd (12.6, 9.1)	1.88, dt (12.9, 5.9)	1.98, dd (13.8, 4.4)
7b	1.67, dd (14.2, 1.3)	1.51, m (12.6)	1.50, ddd (12.9, 5.3, 1.5)	1.17, br, dd (13.8, 4.4)
8a	4.01, d (5.6)	2.05, m	2.18, m	1.70, m
8b		2.03, m	2.05, m	1.64, dt (14.1, 4.4)
9				1.75, m
10a	5.39, q (1.2)	5.59, dt (5.6, 1.5)	5.60, br	1.92, ddd (15.3, 6.5, 3.8)
10b				1.59, m
11		3.64, d (5.6)		3.39, br
13a	3.85, d (12.0)	2.99, d (4.1)	3.89, d (11.4)	3.04, d (4.0)
13b	3.83, d (12.0)	2.80, d (4.1)	3.85, d (11.4)	2.83, d (4.0)
14	0.99, s	0.78, s	1.01, s	0.73, s
15	1.04, s	0.88, s	0.89, s	0.99, s
16a	1.87, d (1.2)	3.97, br	3.98, br	3.74, dd (10.9, 5.6)
16b		3.94, br		3.47, dd (10.9, 5.6)

**Table 2:** ^13^C NMR data (150 Hz, MeOH-*d*_4_) of compounds **1**–**4**.

Position	1	2	3	4
	*δ*_C_, Type	*δ*_C_, Type	*δ*_C_, Type	*δ*_C_, Type
2	81.2, CH	79.3, CH	82.9, CH	79.2, CH
3	40.9, CH_2_	38.4, CH_2_	42.4, CH_2_	38.4, CH_2_
4	74.6, CH	72.5, CH	76.3, CH	72.3, CH
5	54.2, C	48.7, C	55.5, C	49.2, C
6	46.9, C	40.2, C	48.0, C	40.7, C
7	38.9, CH_2_	23.8, CH_2_	31.6, CH_2_	22.7, CH_2_
8	66.7, CH	23.0, CH_2_	25.3, CH_2_	20.9, CH_2_
9	144.7, C	142.9, C	149.5, C	33.9, CH
10	118.2, CH	118.1, CH	116.7, CH	27.9, CH_2_
11	107.5, C	69.9, CH	108.9, C	71.8, CH
12	95.0, C	65.2, C	96.9, C	65.4, C
13	58.1, CH_2_	46.5, CH_2_	59.8, CH_2_	46.9, CH_2_
14	9.6, CH_3_	4.9, CH_3_	14.7, CH_3_	4.6, CH_3_
15	16.0, CH_3_	14.6, CH_3_	10.9, CH_3_	16.3, CH_3_
16	19.1, CH_3_	64.8, CH_2_	65.6, CH_2_	64.3, CH_2_

**Table 3:** IC_50_ values of compounds **1**–**9** against three cancer cell lines (HCT-116, PC-3, and SK-Hep-1), inhibition of NO production in microglial BV-2 cell-induced LPS treated with 10 μM of compounds **1**–**9**, and percent cell viability in BV-2 cell.

Compounds	Cytotoxicity (IC_50_, μM)			NO (μM) ± SD	Cell Viability (%) ± SD in BV-2 Cell
	HCT-116	PC-3	SK-Hep-1		
**1**	>10	>10	>10	10.8 ± 2.1 ***	95.4 ± 5.7
**2**	>10	>10	>10	8.1 ± 0.7 ***	99.9 ± 1.6
**3**	>10	>10	>10	12.4 ± 1.7 **	105.8 ± 2.9
**4**	>10	>10	>10	9.2 ± 1.2 ***	102.8 ± 9.4
**5**	5.4 ± 0.3	6.4 ± 0.1	5.0 ± 0.3	1.9 ± 0.5 ***	62.9 ± 3.7 ***
**6**	>10	>10	>10	12.1 ± 1.5 **	104.9 ± 12.2
**7**	7.5 ± 0.3	9.3 ± 0.4	5.9 ± 0.2	4.2 ± 1.1 ***	94.7 ± 17.8
**8**	3.3 ± 0.3	5.3 ± 0.3	1.8 ± 0.8	1.9 ± 0.1 ***	63.3 ± 6.4 ***
**9**	>10	>10	>10	12.5 ± 0.8 **	103.5 ± 4.5
Trichodermin ^a^	0.5 ± 0.0	0.9 ± 0.1	0.4 ± 0.1	-	-
Resting	-	-	-	2.4 ± 0.2	100 ± 0.0
Vehicle	-	-	-	16.4 ± 0.5 ^###^	-

^a^ Positive control for cytotoxicity test; ** *p* < 0.01, and *** *p* < 0.001 compared with the vehicle; ^###^ *p* < 0.001 compared with the resting.

## Data Availability

Not applicable.

## Supplementary Materials

The following are available online at https://www.mdpi.com/article/10.3390/md20020080/s1, Figure S1: 1H NMR (600 MHz, MeOH-d4) of **1**, Figure S2: 13C NMR (150 MHz, MeOH-d4) of **1**, Figure S3: DEPT of **1**, Figure S4: HSQC of **1**, Figure S5: HMBC of **1**, Figure S6: COSY of **1**, Figure S7: NOESY of **1**, Figure S8: 1H NMR (600 MHz, MeOH-d4) of **2**, Figure S9: 13C NMR (150 MHz, MeOH-d4) of **2**, Figure S10: DEPT of **2**, Figure S11: HSQC of **2**, Figure S12: HMBC of **2**, Figure S13: COSY of **2**, Figure S14: NOESY of **2**, Figure S15: 1H NMR (600 MHz, MeOH-d4) of **3**, Figure S16: 13C NMR (150 MHz, MeOH-d4) of **3**, Figure S17: DEPT of **3**, Figure S18: HSQC of **3**, Figure S19: HMBC of **3**, Figure S20: COSY of **3**, Figure S21: NOESY of **3**, Figure S22: 1H NMR (600 MHz, MeOH-d4) of **4**, Figure S23: 13C NMR (150 MHz, MeOH-d4) of **4**, Figure S24: DEPT of **4**, Figure S25: HSQC of **4**, Figure S26: HMBC of **4**, Figure S27: COSY of **4**, Figure S28: NEOSY of **4**, Figure S29: MS spectrum of **1**, Figure S30: MS spectrum of **2**, Figure S31: MS spectrum of **3**, Figure S32: MS spectrum of **4**, Figure S33: IR spectrum of **1,** Figure S34: IR spectrum of **2**, Figure S35: IR spectrum of **3**, Figure S36: IR spectrum of **4**, Figure S37: Cell viability in human THP-1 monocytic cells, Physical data of the new compounds.

## Abbreviations

NMR	nuclear magnetic resonance
MS	mass spectrometry
IR	infrared spectroscopy
HCT	human colorectal carcinoma cell
PC	human prostate cell
SK-Hep	human hepatic carcinoma cell
SBR	sulforhodamine B
LPS	lipopolysaccharide
NO	nitric oxide
MMP	matrix metalloproteinase
THP-1	human leukemia monocytic cell line
COSY	correlation spectroscopy
NOESY	nuclear overhauser effect spectroscopy
