# Supplementary material for: New Trichothecenes Isolated from the Marine Algicolous Fungus Trichoderma brevicompactum
Source: Mar Drugs. 2022 Jan 18;20(2):80. doi: 10.3390/md20020080 (PMC8875558; doi:10.3390/md20020080)
Supplement: Supplementary file 1 [file marinedrugs-20-00080-s001.zip › marinedrugs-1509869-supplementary.pdf]

Supporting Information
for

New Trichothecenes Isolated from the Marine Alga Endophytic Fungus *Trichoderma brevicompactum*

Safwan Safwan, Shih-Wei Wang, George Hsiao, Sui-Wen Hsiao, Su-Jung Hsu, Tzong-Huei Lee and Ching-Kuo Lee

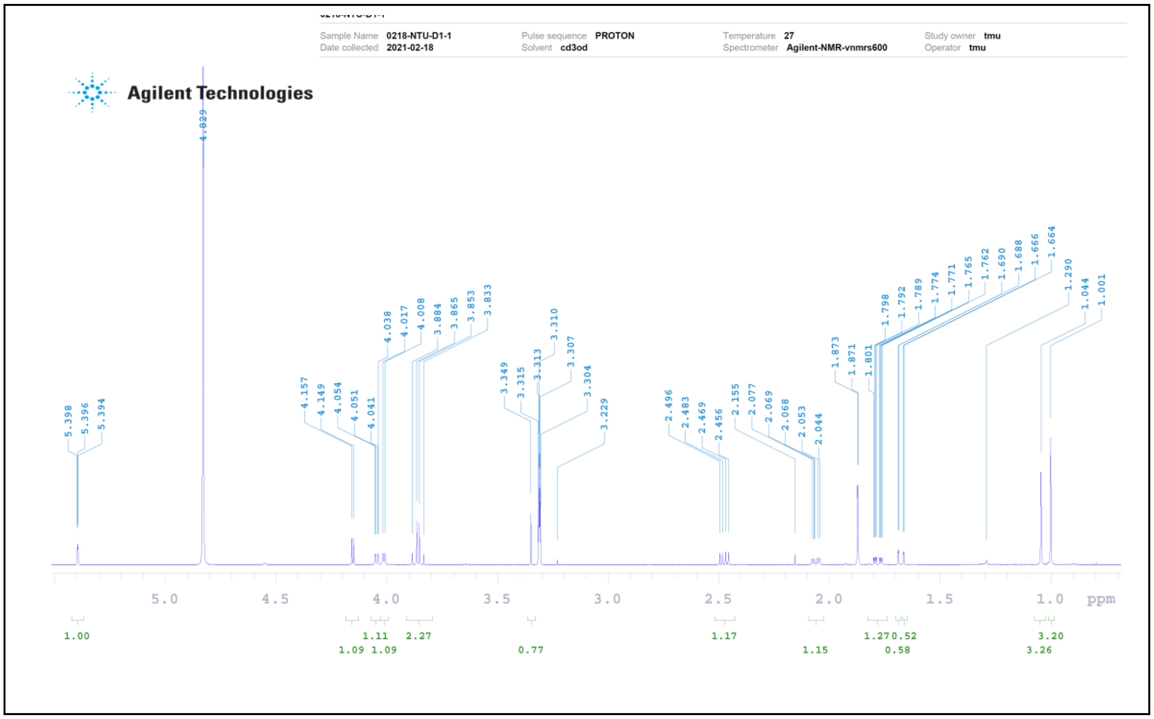

Figure S1. ^{1}H NMR (600 MHz, MeOH-d_4) of **1**

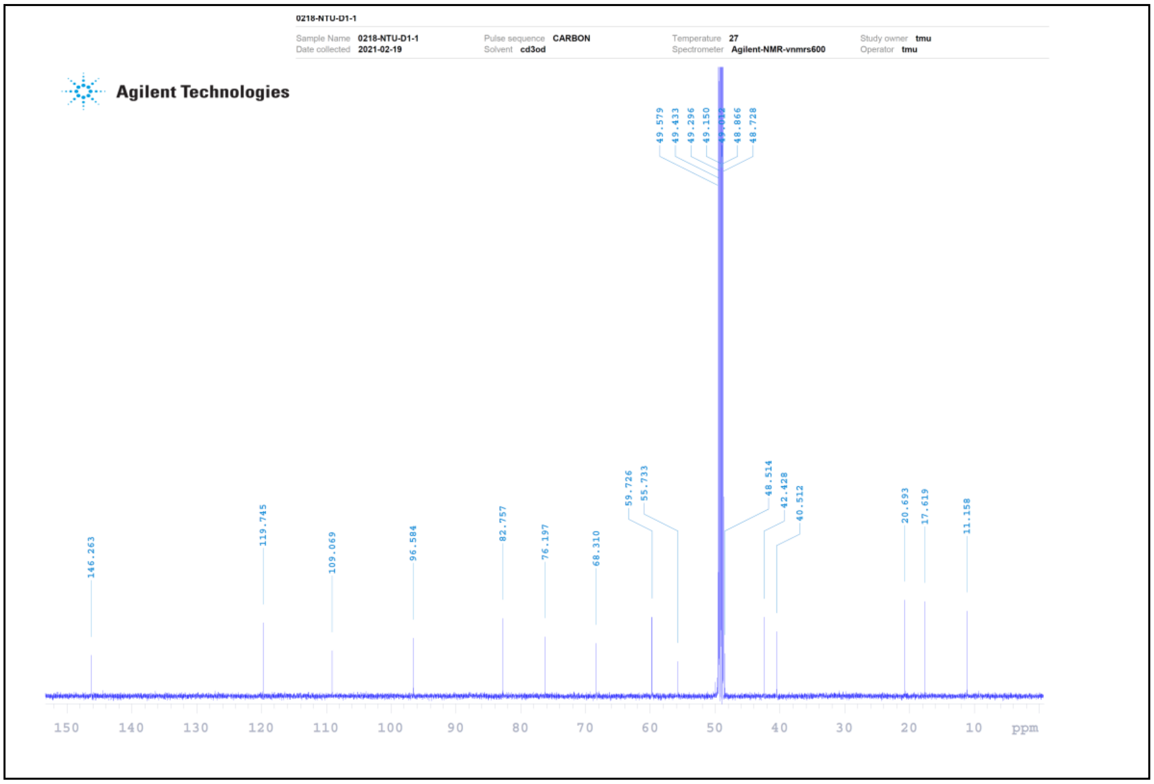

Figure S2. ^{13}C NMR (150 MHz, MeOH-d_4) of **1**

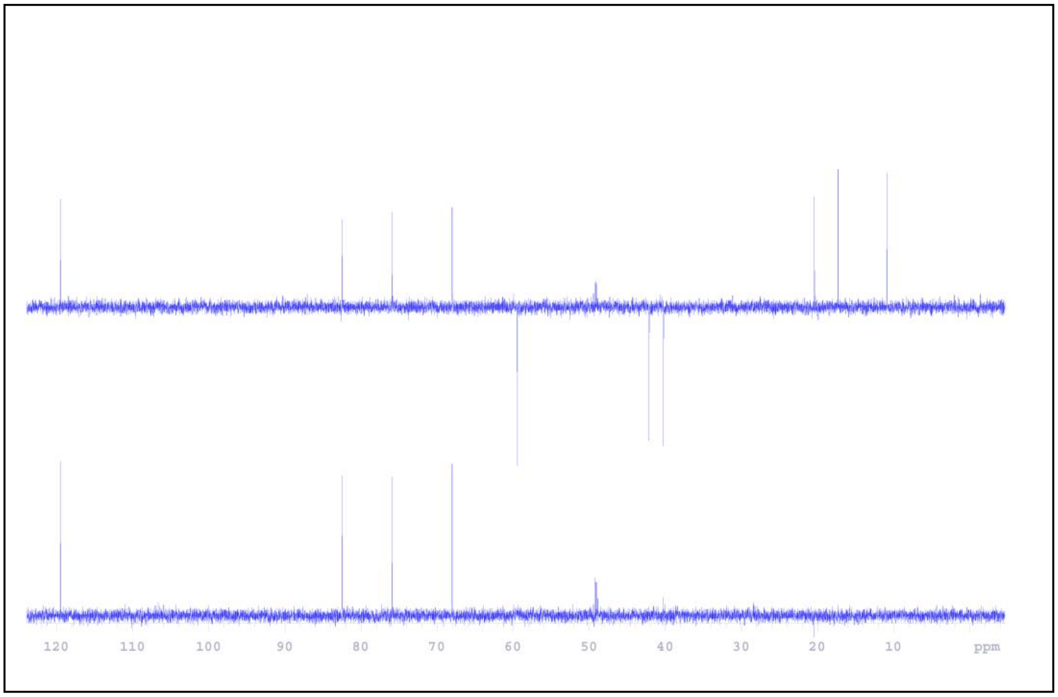

Figure S3.DEPT -90 (down) and -135(up) of **1**.

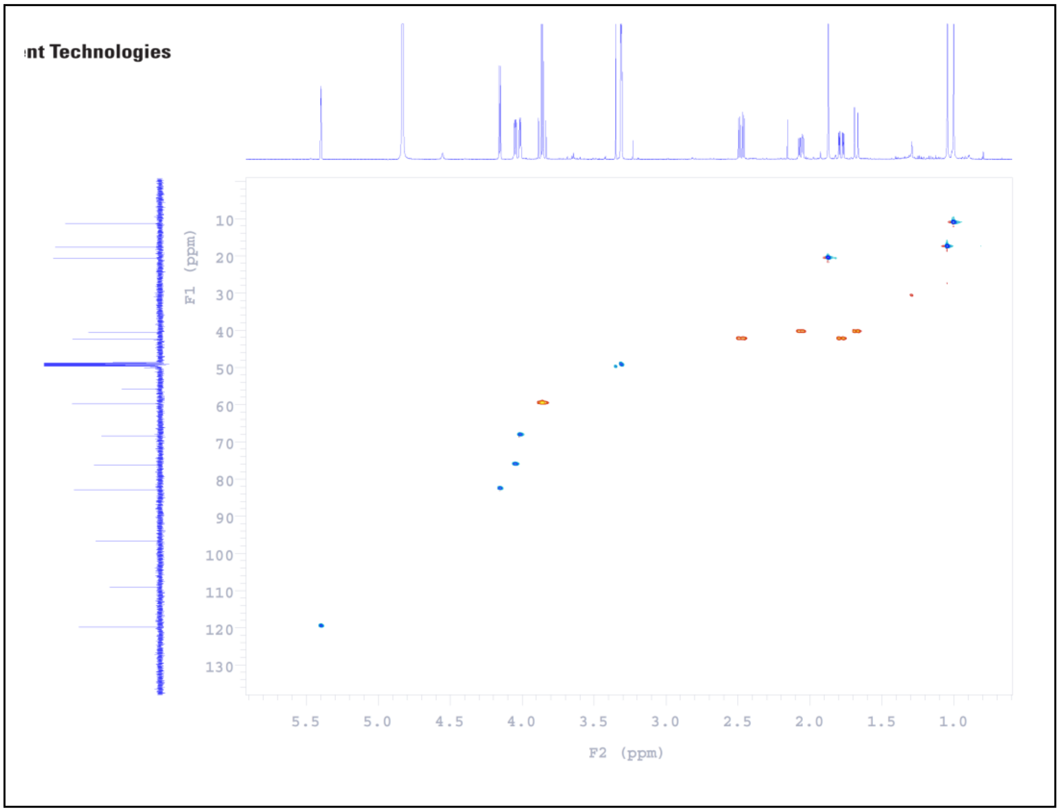

Figure S4. HSQC of **1**.

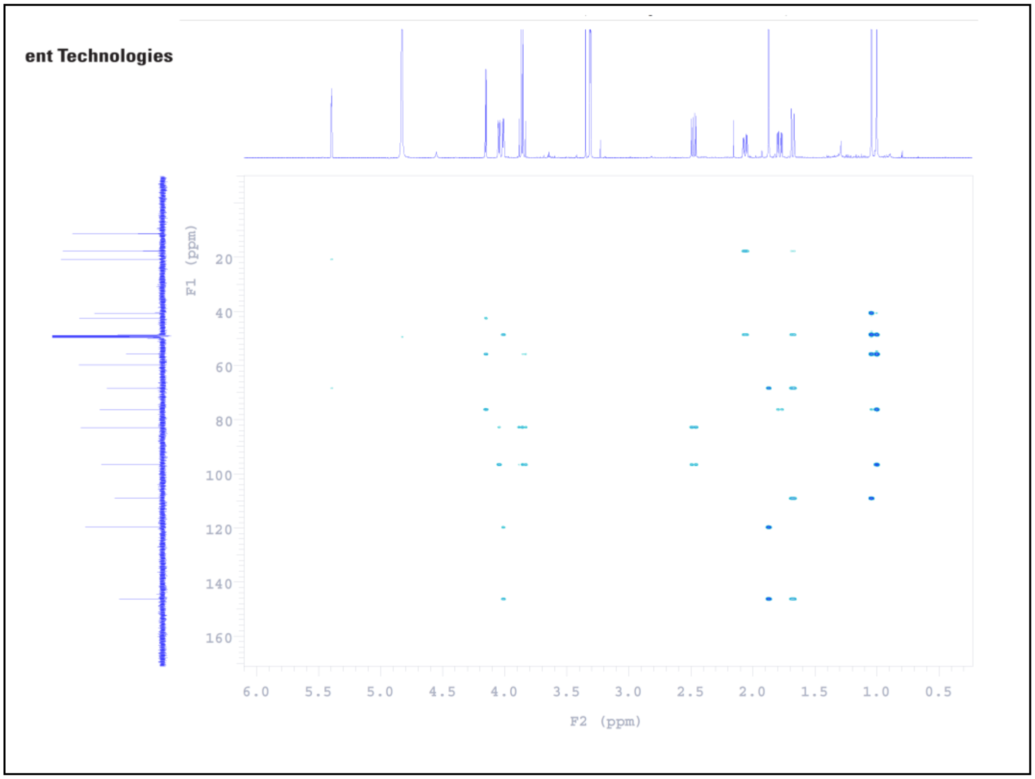

Figure S5. HMBC of **1**.

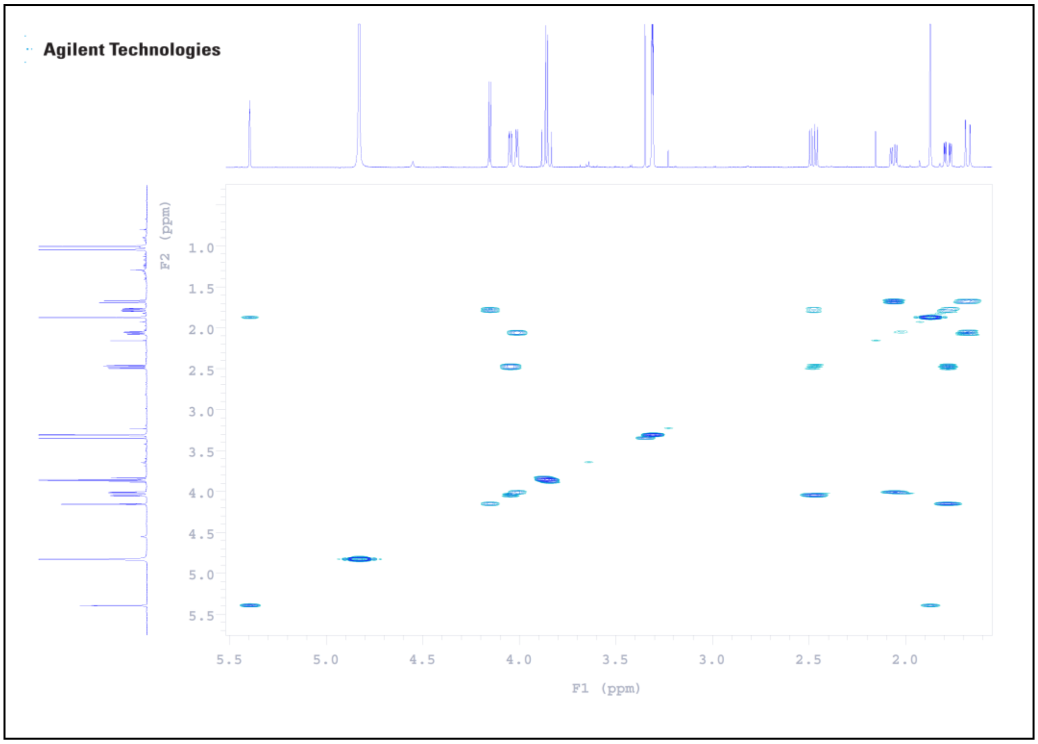

Figure S6. COSY of **1**.

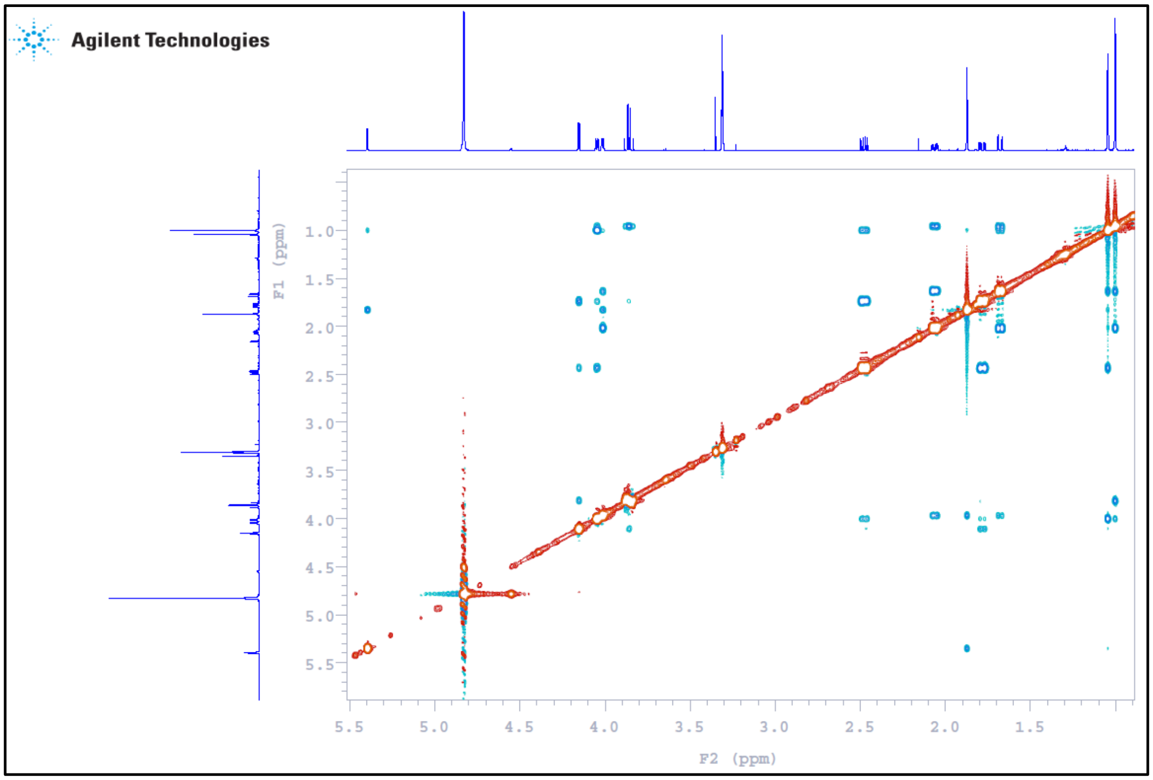

Figure S7. NOESY of **1**.

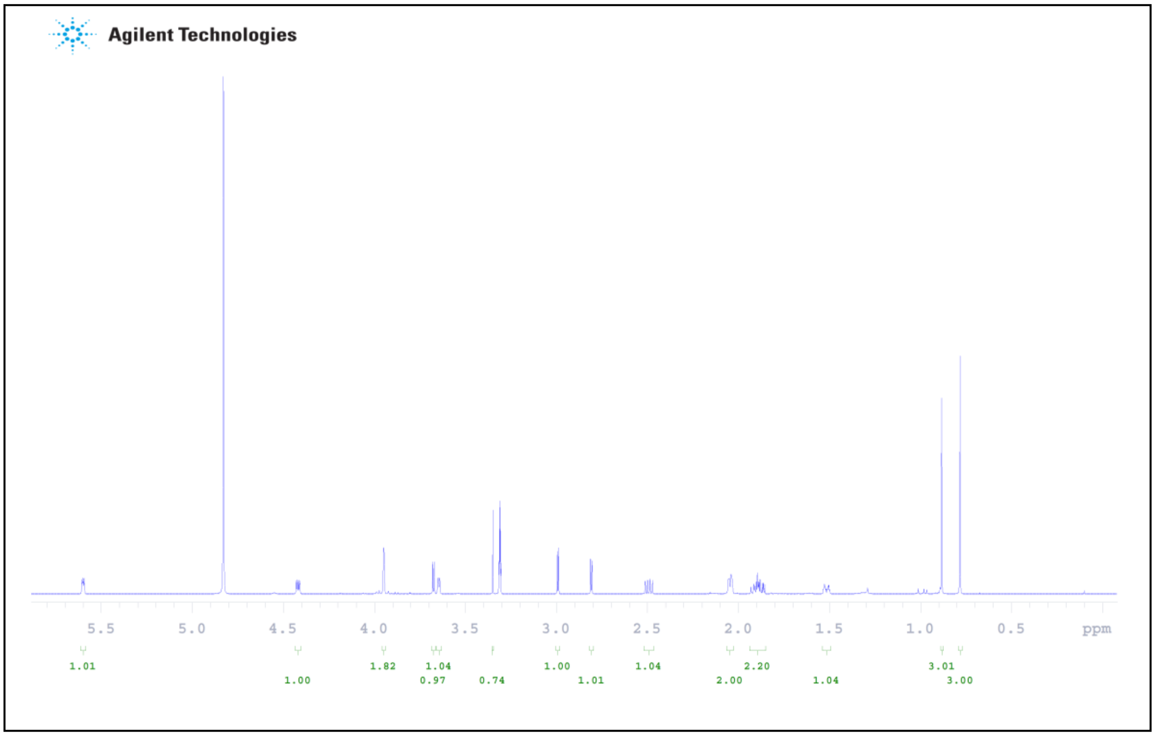

Figure S8. ^{1}H NMR (600 MHz, MeOH-d_4) of **2**

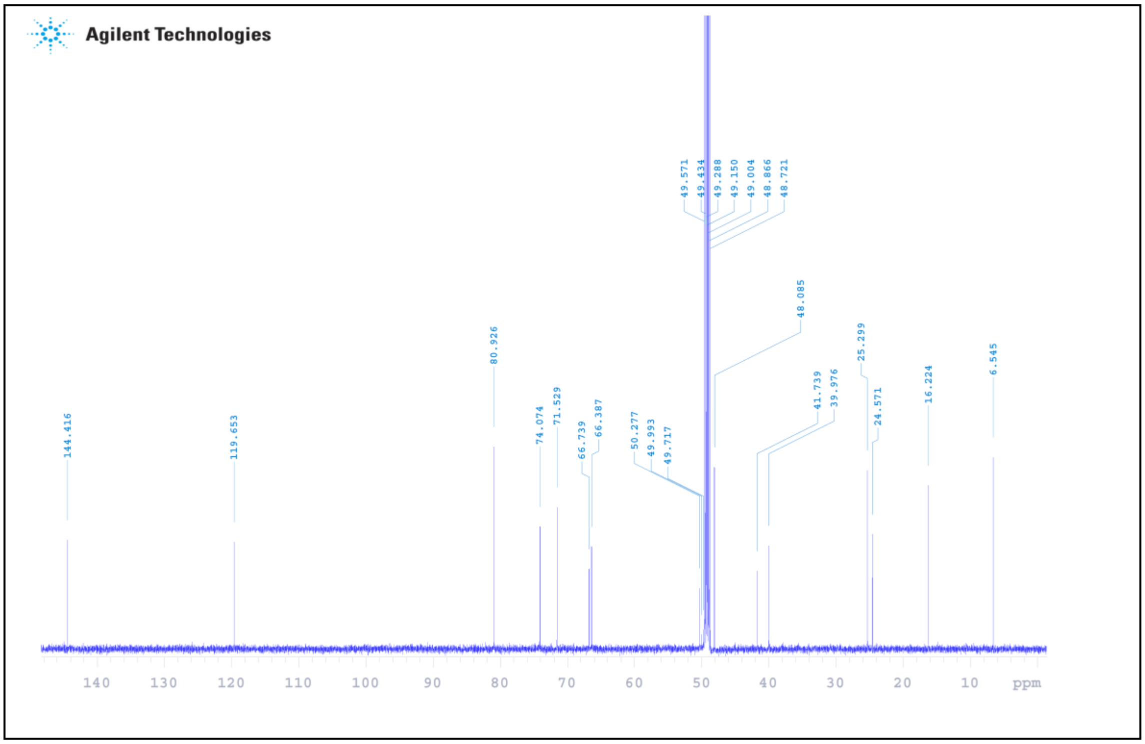

Figure S9. ^{13}C NMR (150 MHz, MeOH-d_4) of **2**.

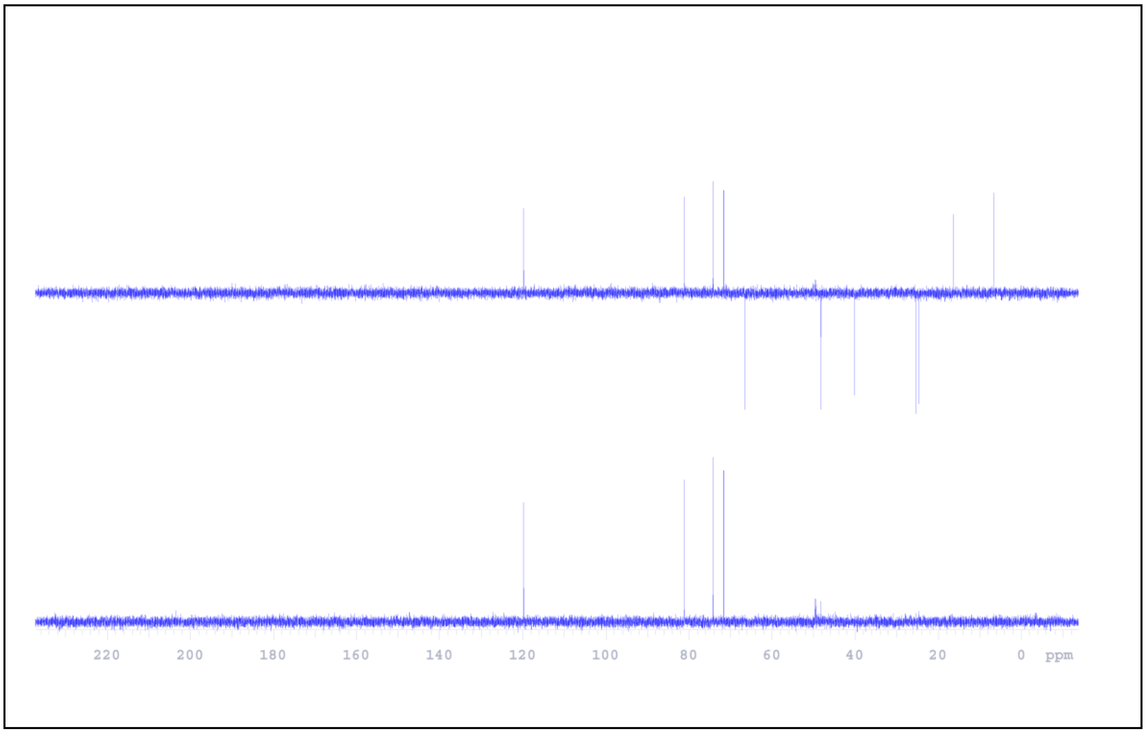

Figure S10. DEPT -90 (down) and -135(up) of **2**

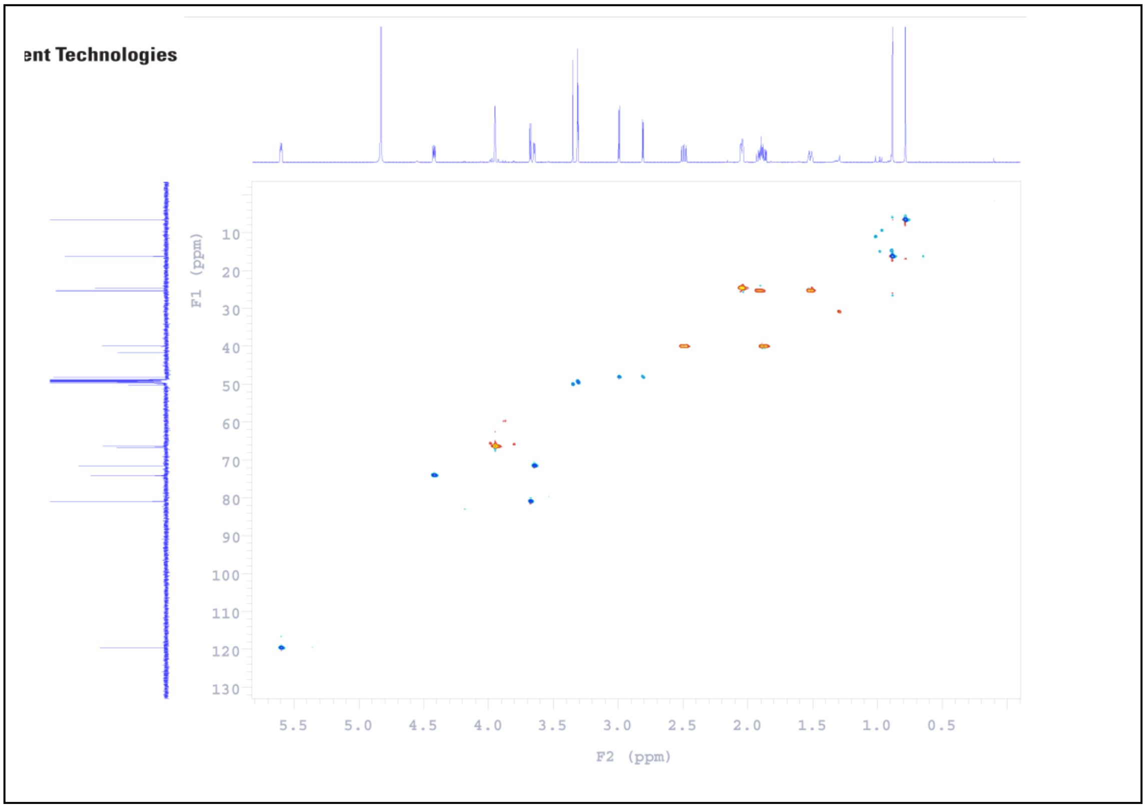

Figure S11. HSQC of **2**

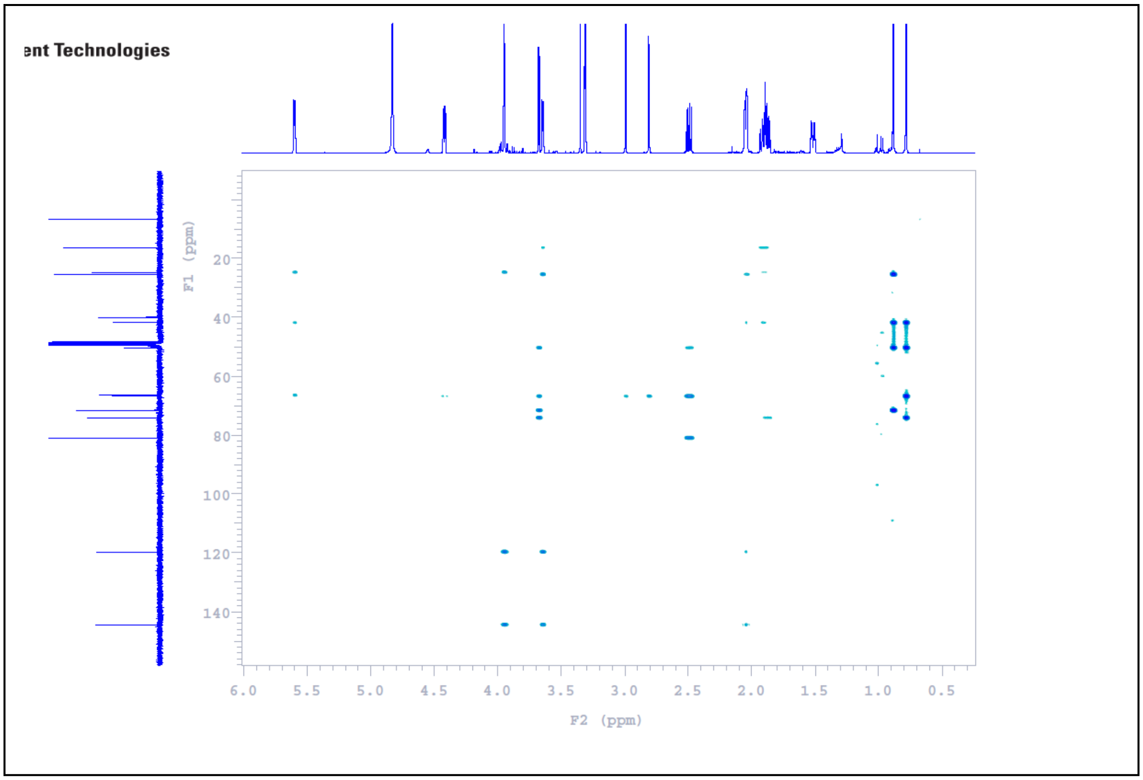

Figure S12. HMBC of **2**.

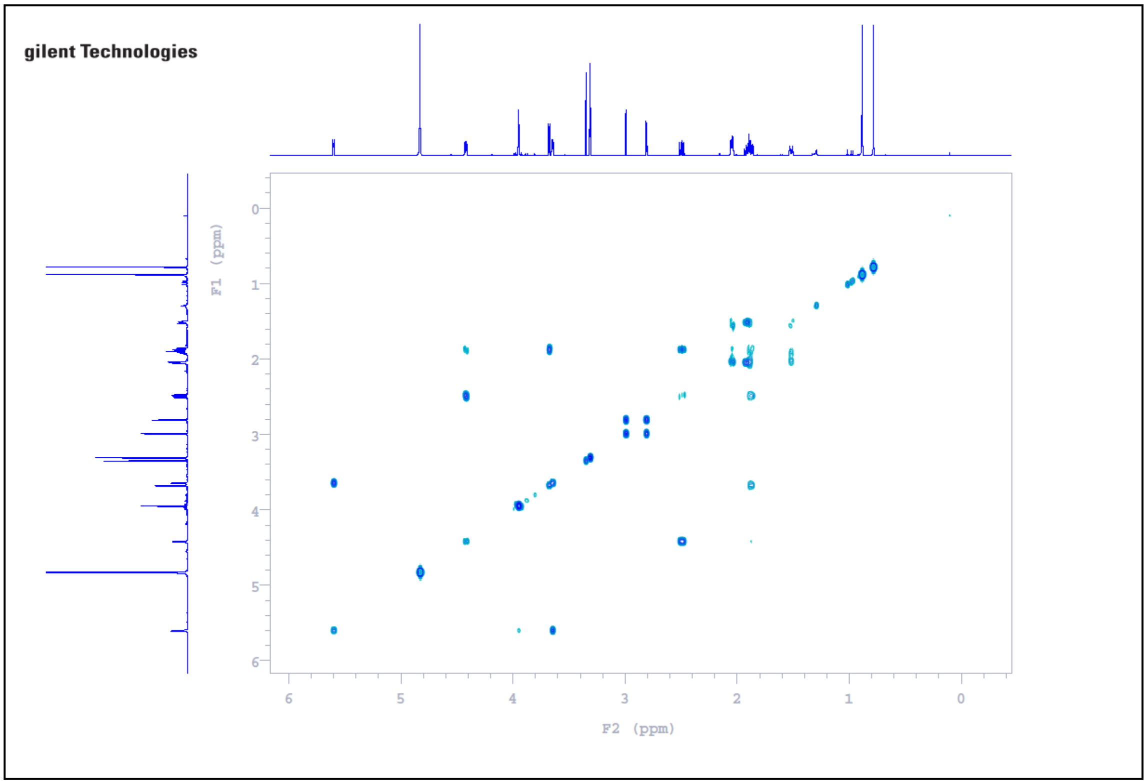

Figure S13. COSY of **2**.

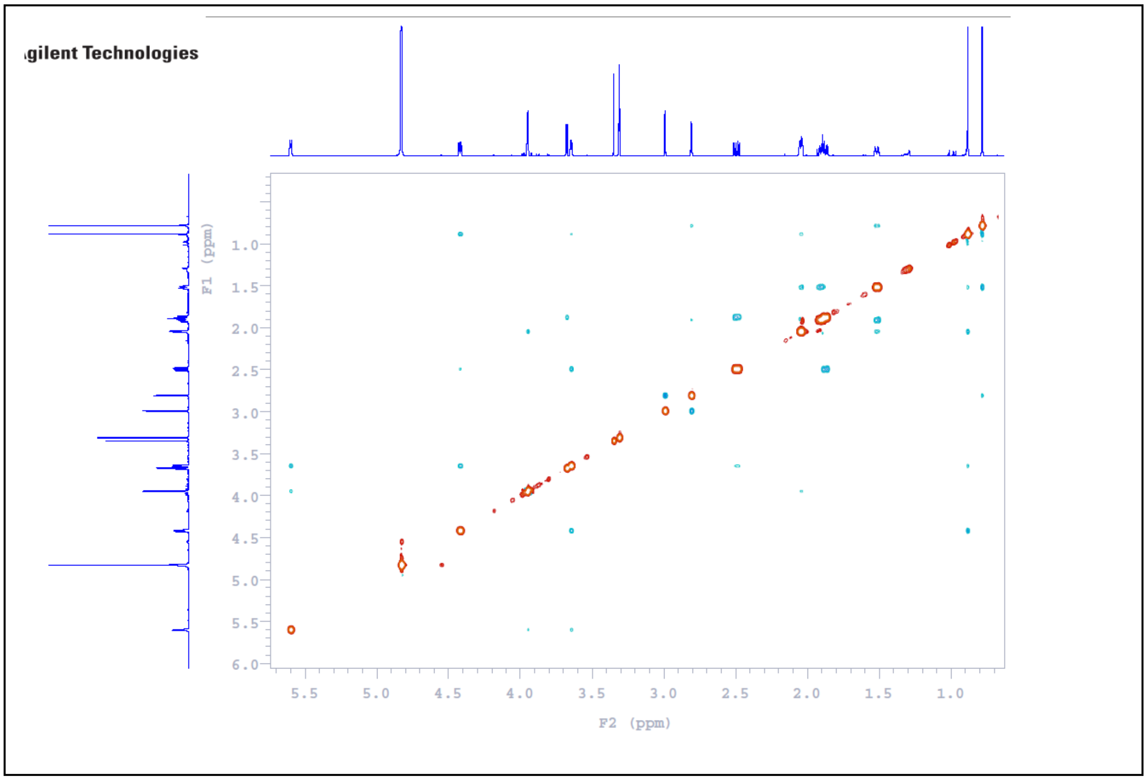

Figure S14. NOESY of **2**.

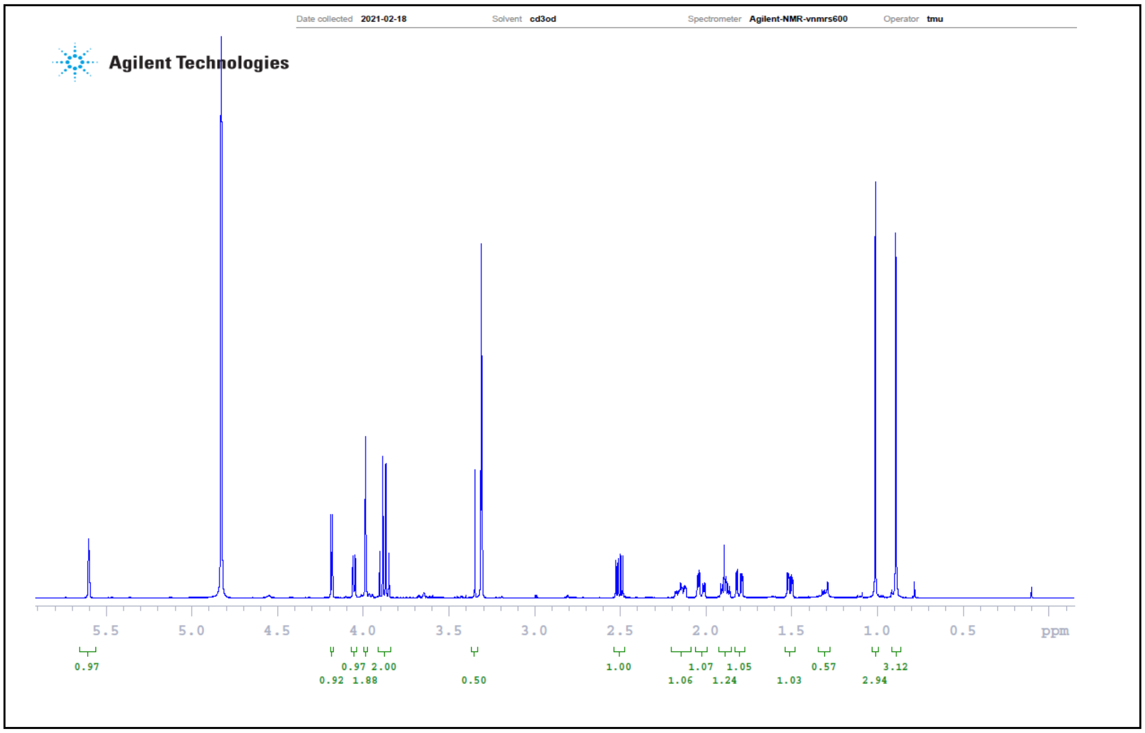

Figure S15. ^{1}H NMR (600 MHz, MeOH-d_4) of **3**

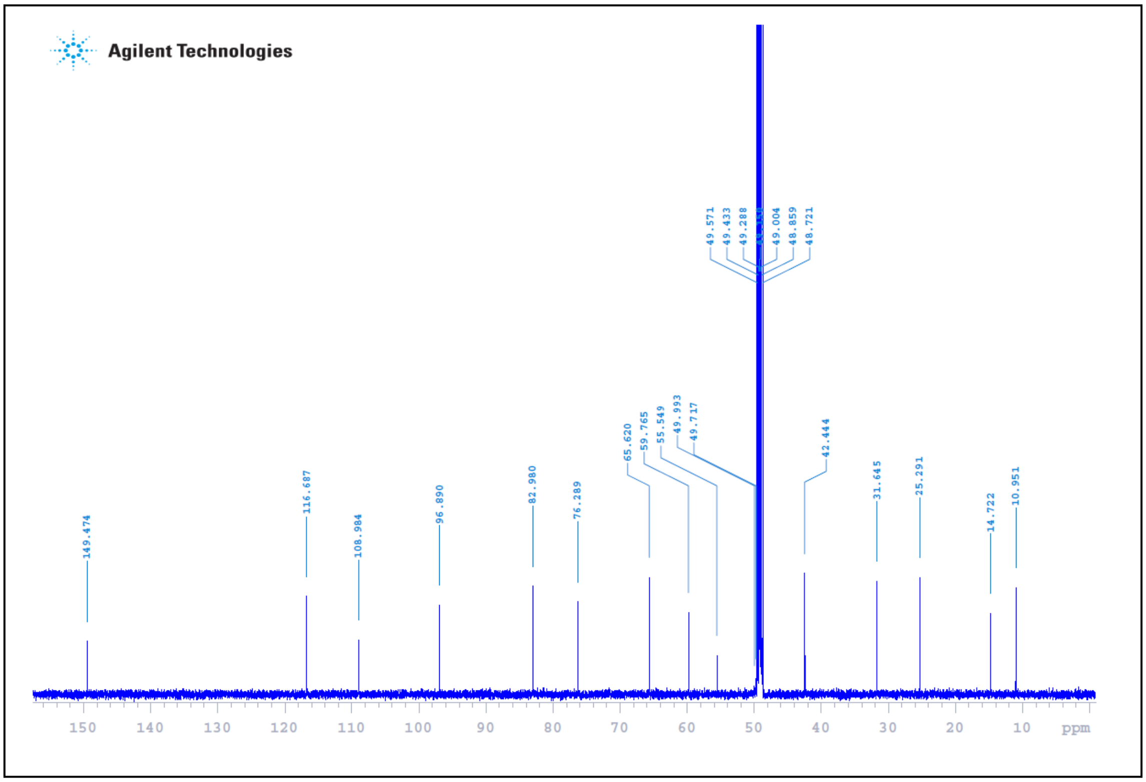

Figure S16. ^{13}C NMR (150 MHz, MeOH-d_4) of **3**

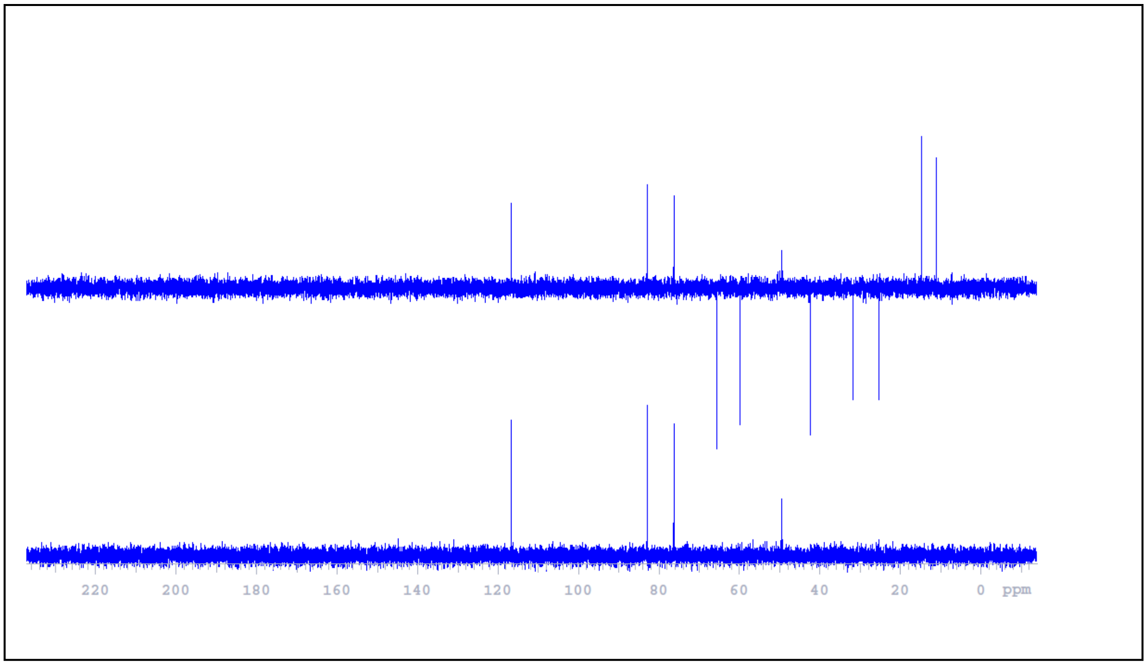

Figure S17. DEPT -90 (down) and -135(up) of **3**.

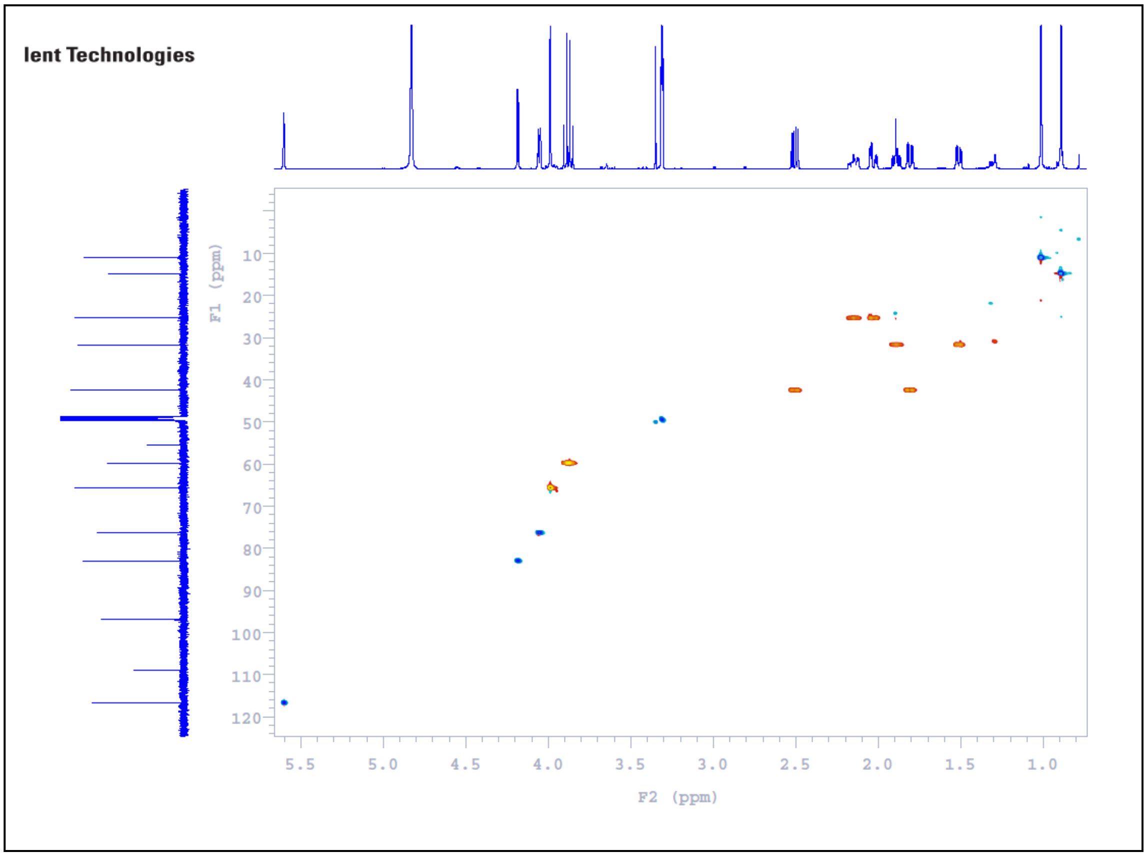

Figure S18. HSQC of **3**.

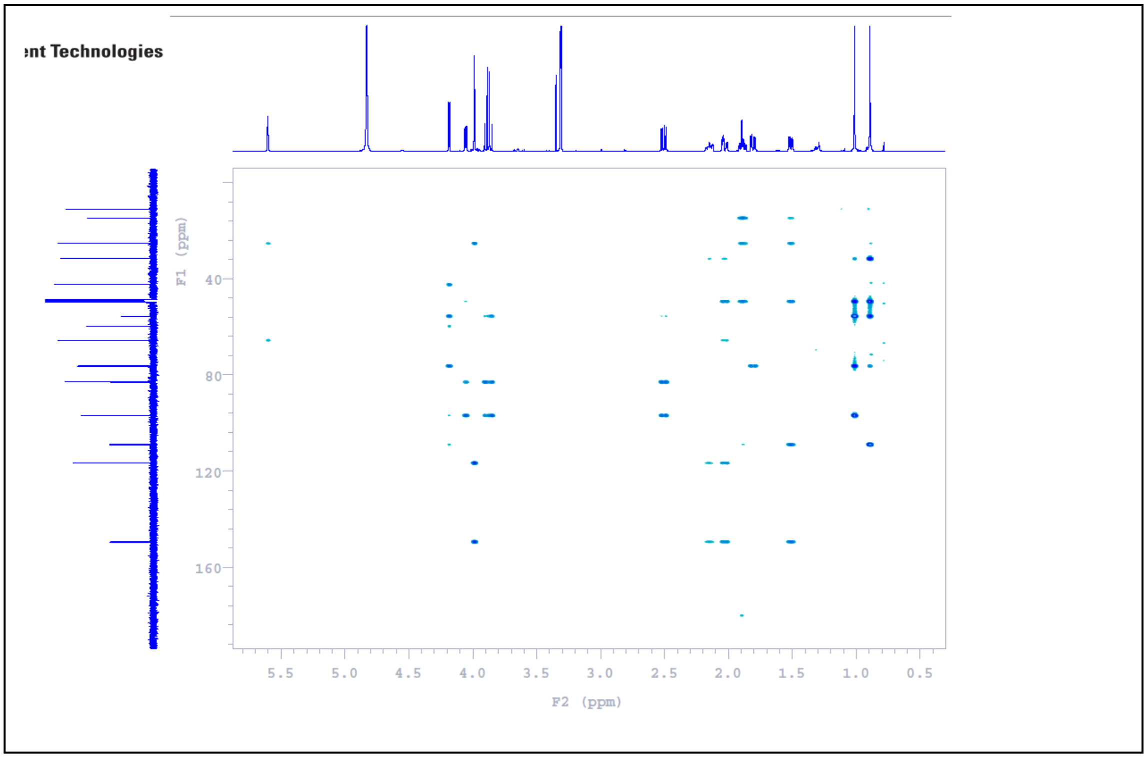

Figure S19. HMBC of **3**.

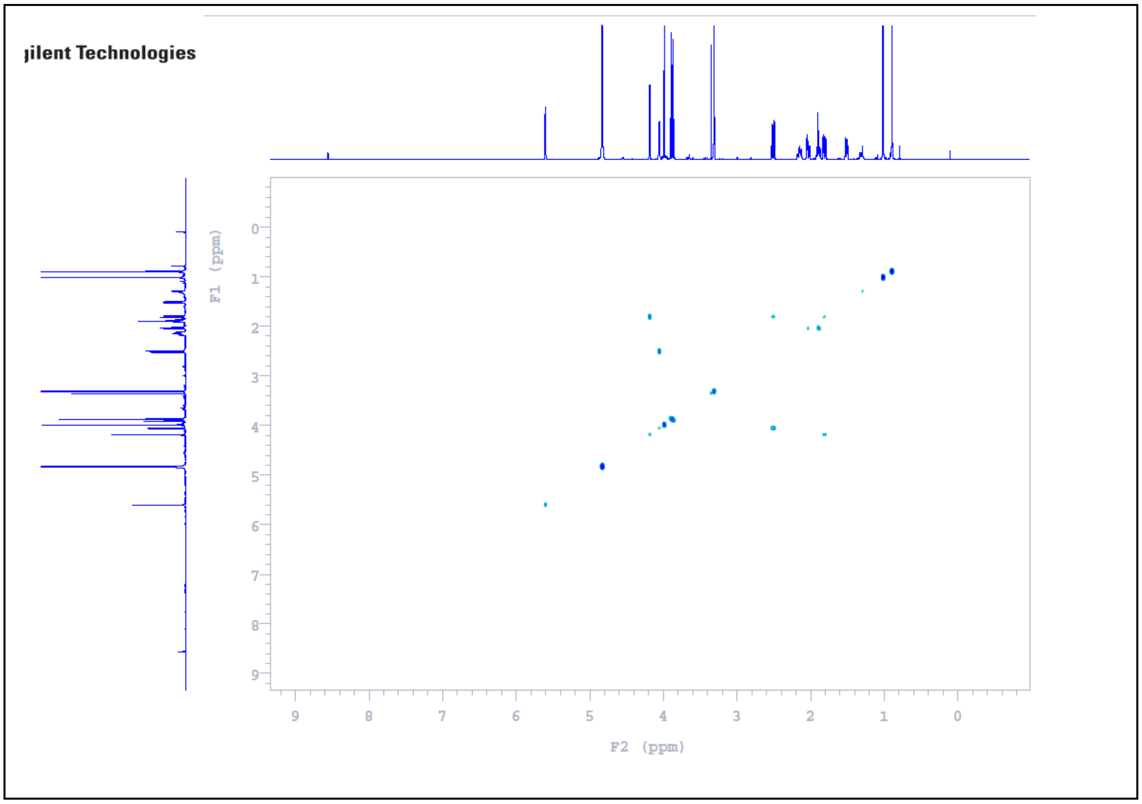

Figure S20. COSY of **3**.

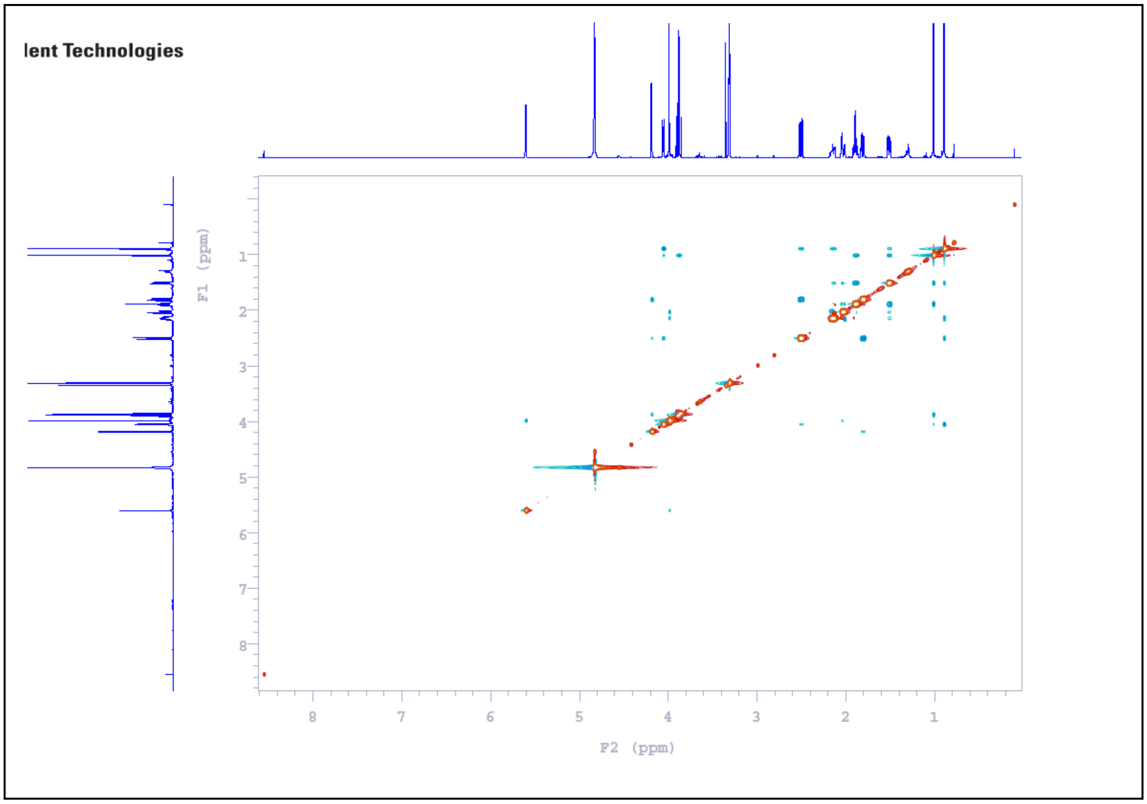

Figure S21. NOESY of **3**.

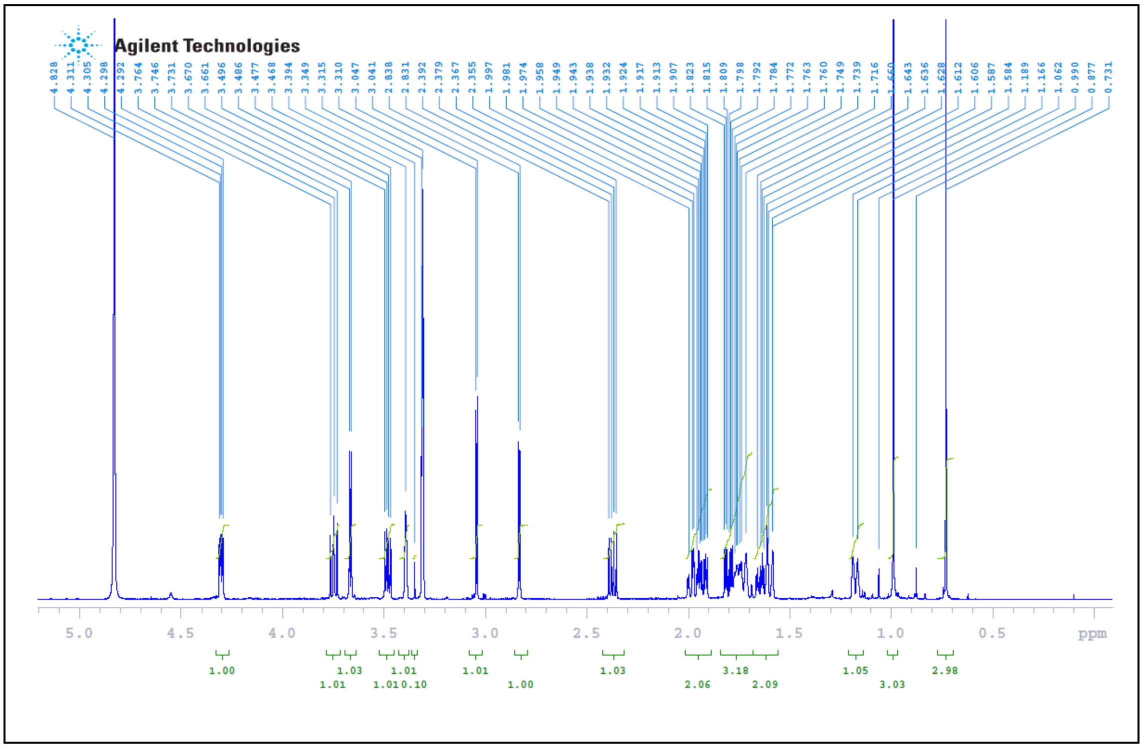

Figure S22. ^{1}H NMR (600 MHz, MeOH-d_4) of **4**.

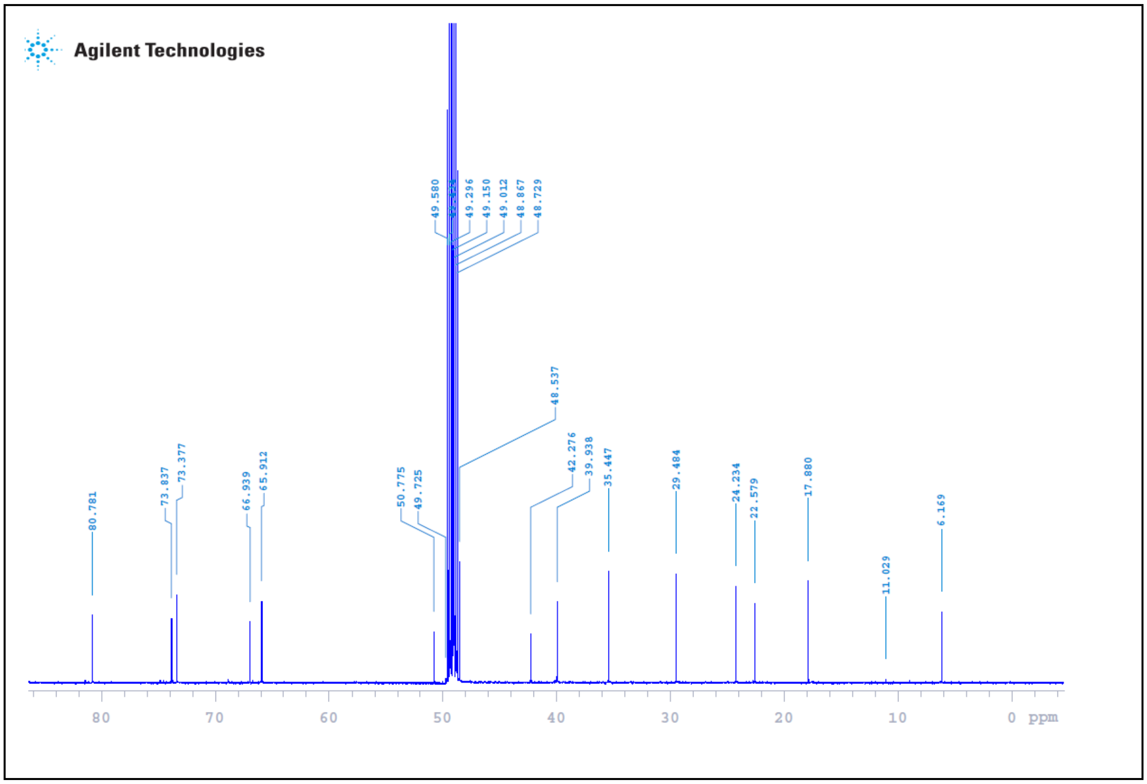

Figure S23. ^{13}C NMR (150 Hz, MeOH-d_4) of **4**.

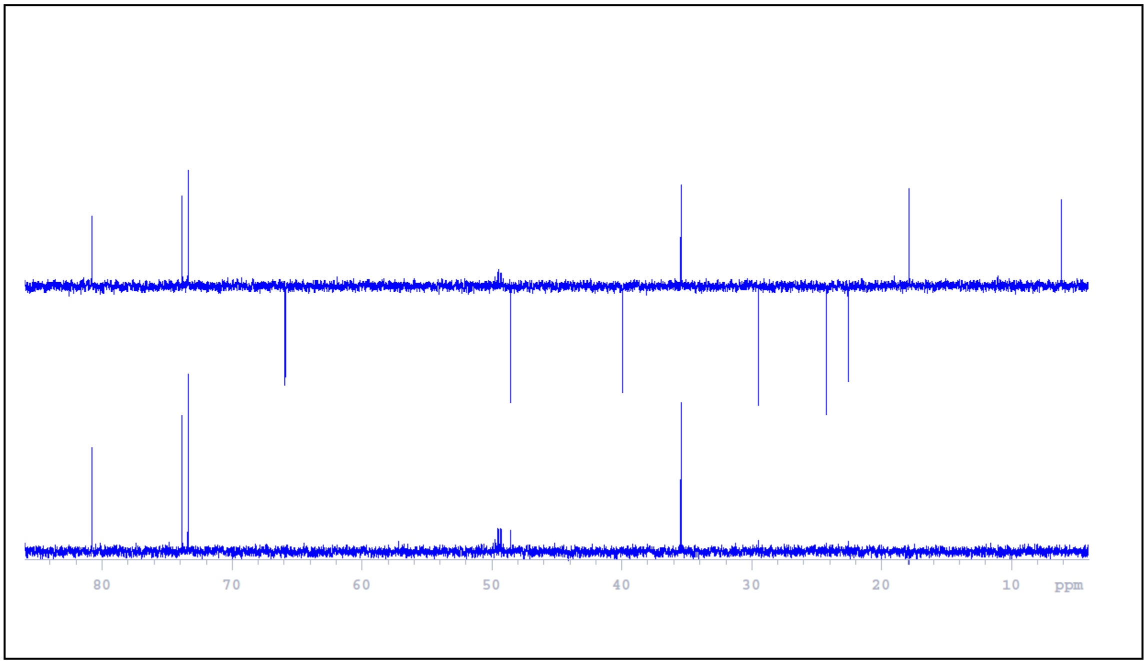

Figure S24. DEPT -90 (down) and -135(up) of **4**.

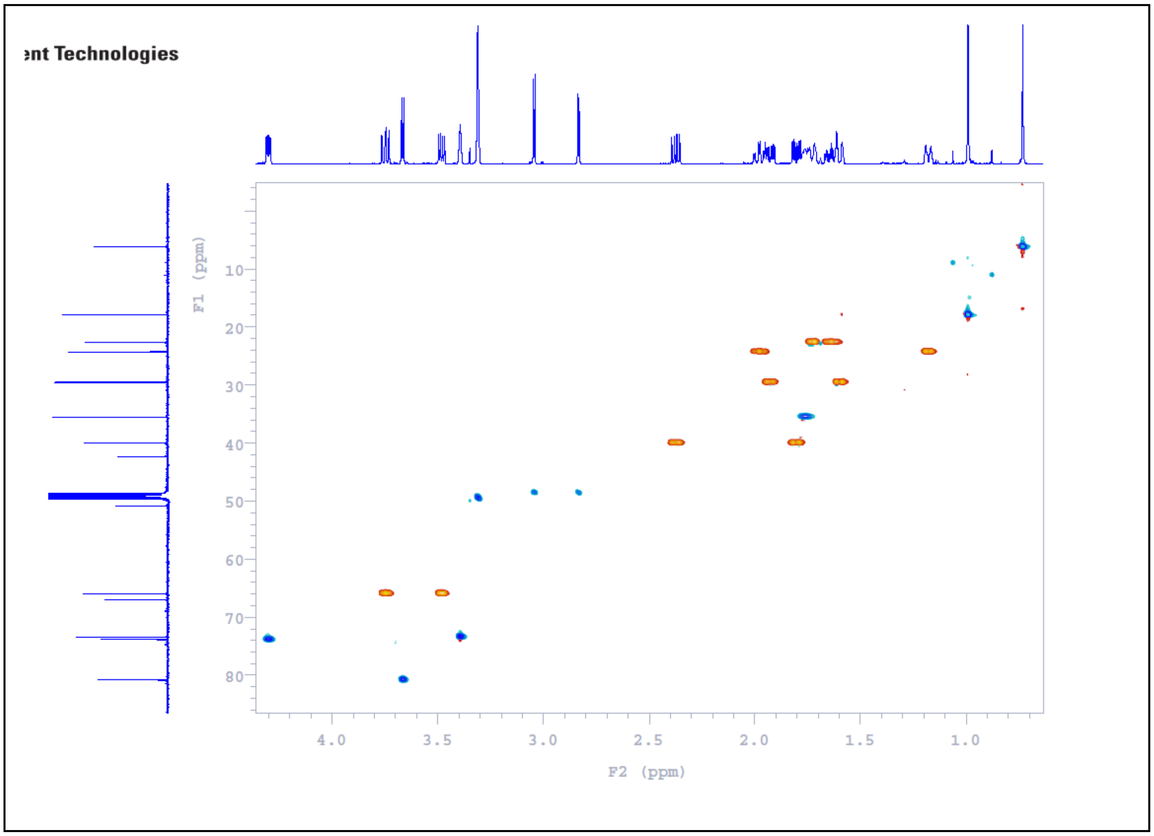

Figure S25. HSQC of **4**.

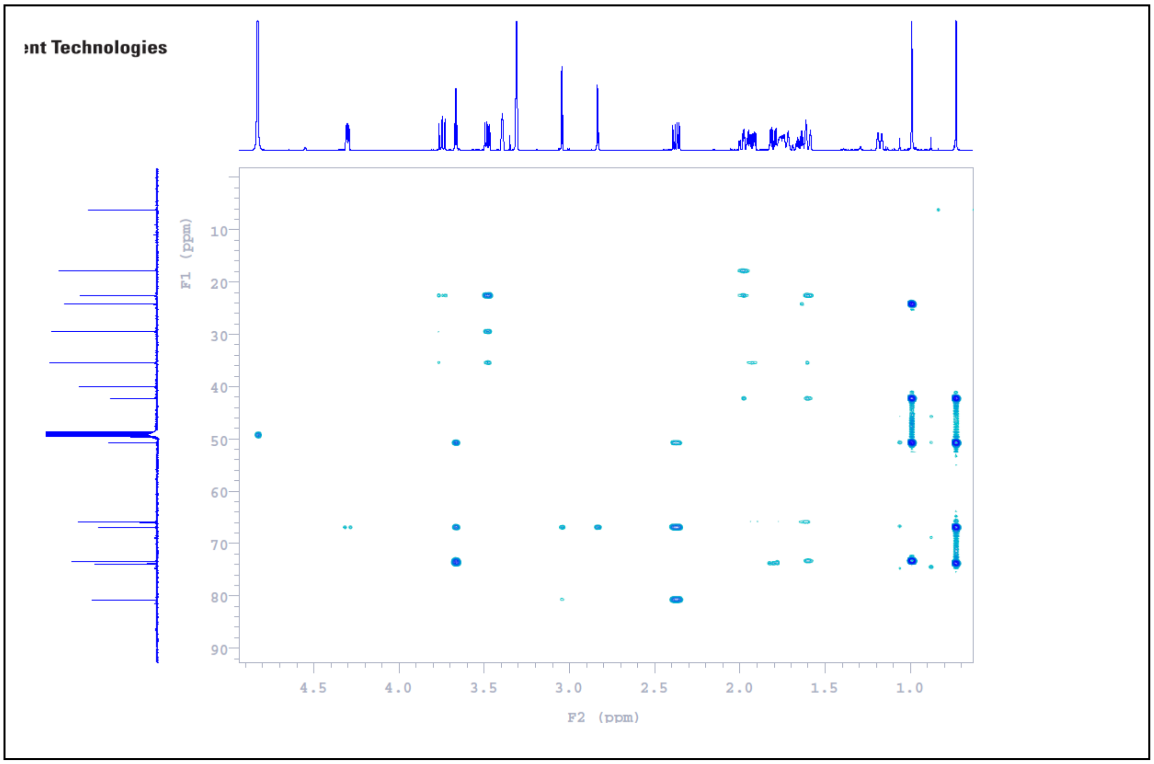

Figure S26. HMBC of **4**.

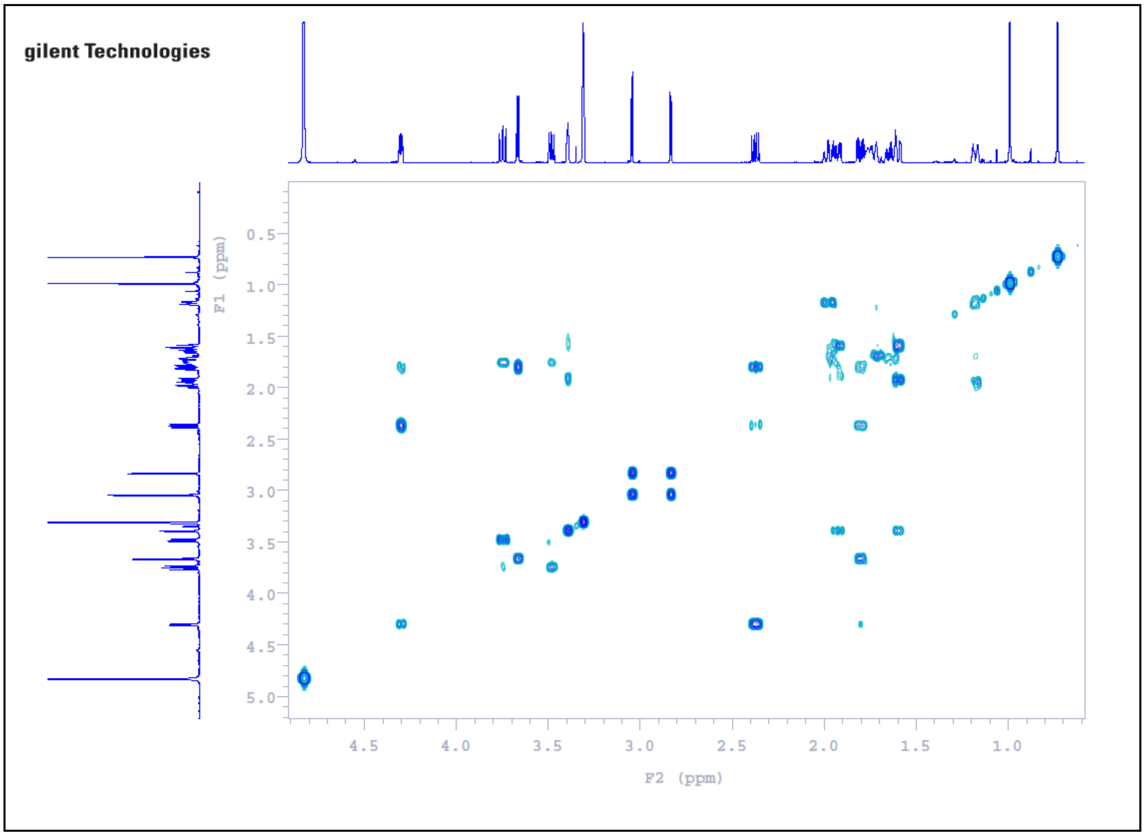

Figure S27. COSY of **4**.

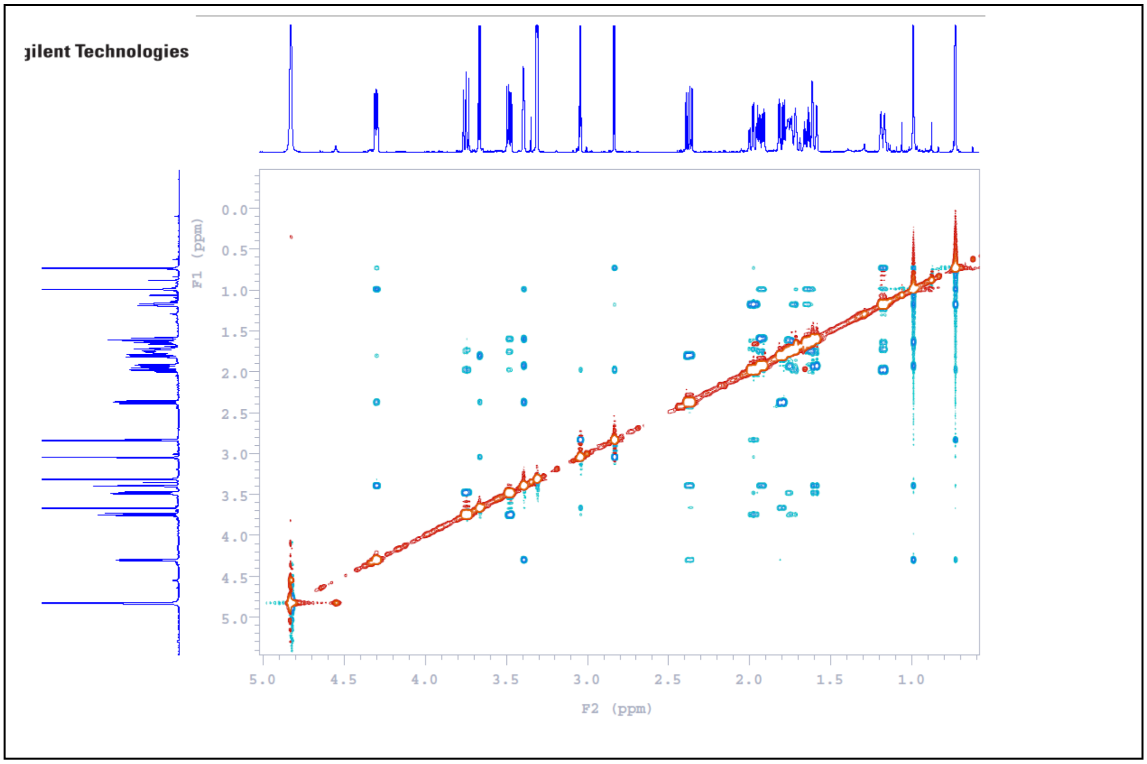

Figure S28. NEOSY of **4**.

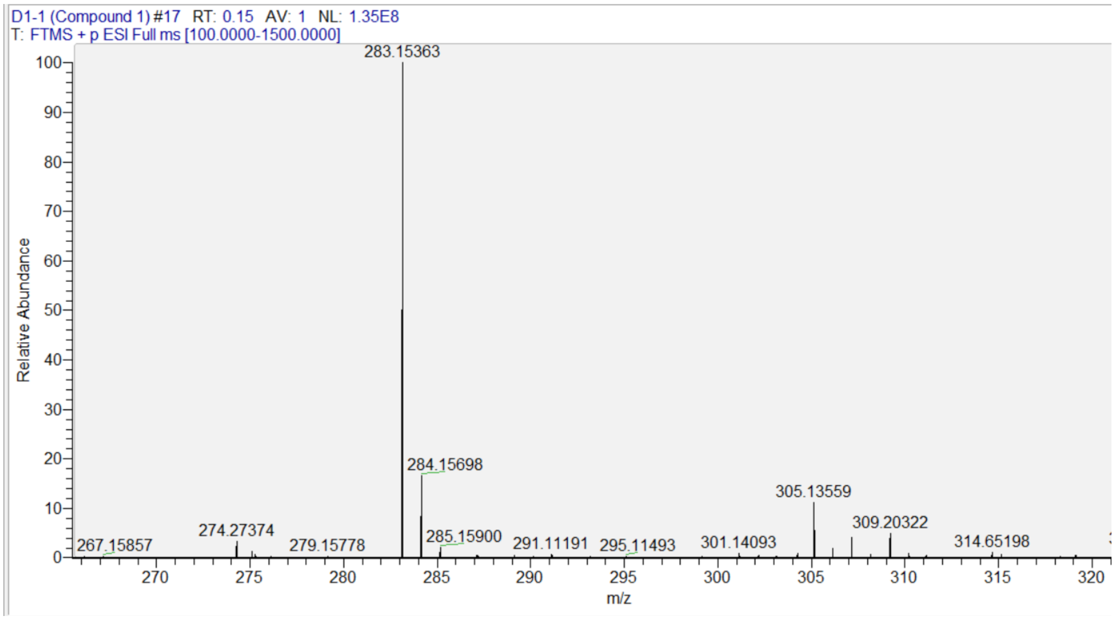

Figure S29. MS spectrum of **1**.

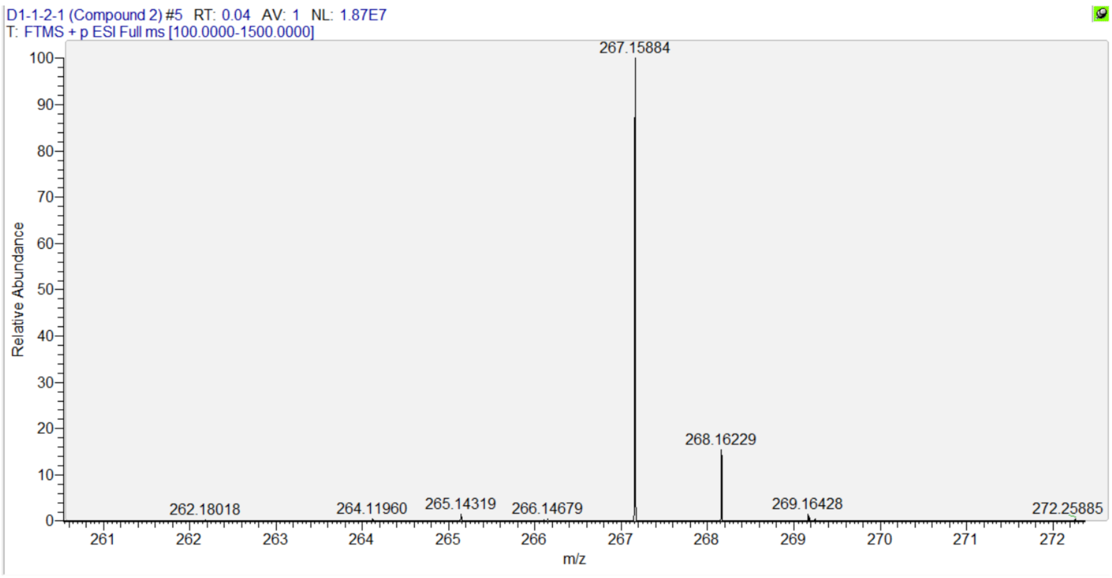

Figure S30. MS spectrum of **2**.

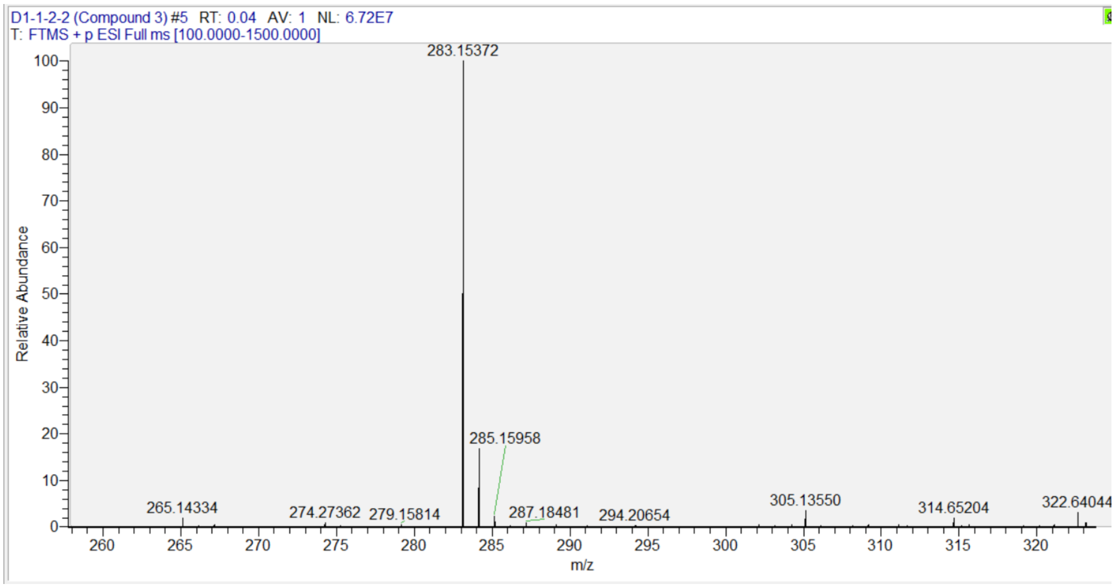

Figure S31. MS spectrum of **3**.

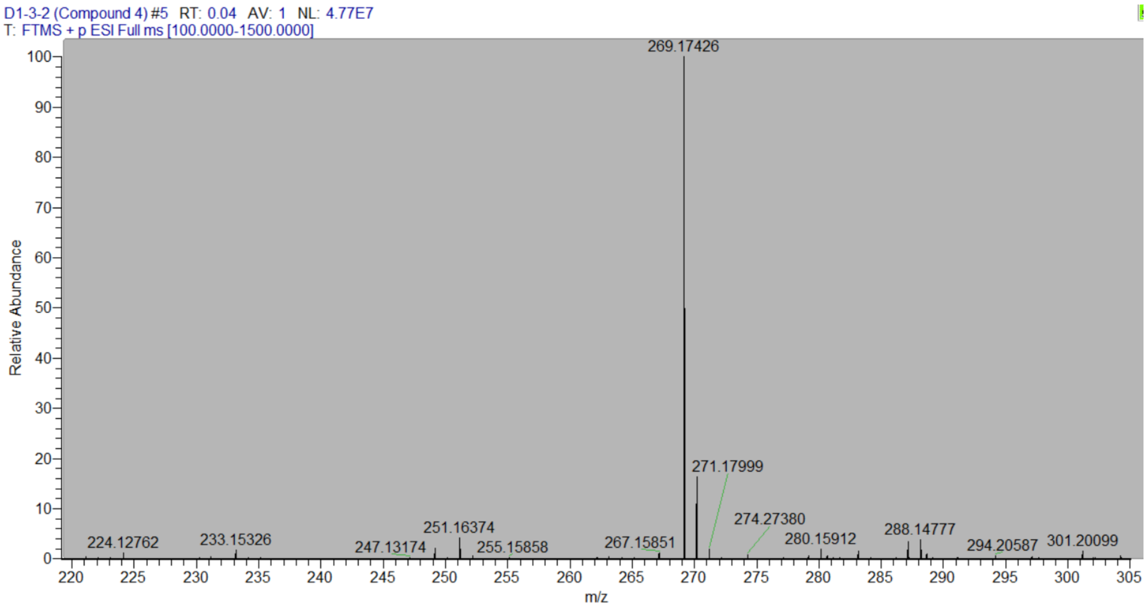

Figure S32. MS spectrum of **4**.

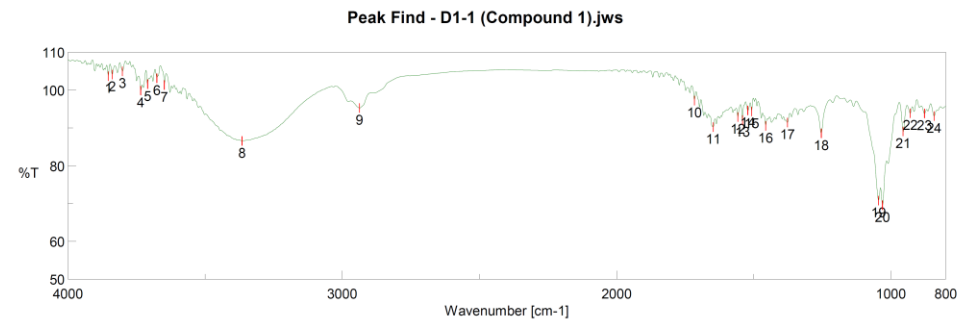

[Result of Peak Picking]

No.	Position	Intensity	No.	Position	Intensity	No.	Position	Intensity
1	3853.08	103.643	2	3838.61	104.074	3	3801.01	104.932
4	3734.48	99.8654	5	3709.41	101.565	6	3675.66	103.087
7	3648.66	101.282	8	3365.17	86.5295	9	2937.06	95.2117
10	1716.34	97.1816	11	1646.91	90.183	12	1558.2	92.8367
13	1540.85	92.0462	14	1521.56	94.5683	15	1507.1	94.2975
16	1456.96	90.4901	17	1376.93	91.3743	18	1253.5	88.5298
19	1045.23	70.7548	20	1030.77	69.5115	21	955.555	89.0511
22	929.521	93.8378	23	877.452	93.7692	24	842.74	93.1124

Figure S33. IR spectrum of **1**.

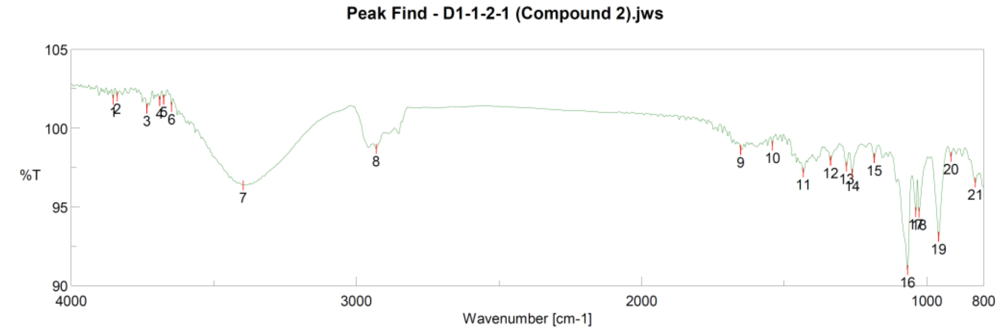

[Result of Peak Picking]

No.	Position	Intensity	No.	Position	Intensity	No.	Position	Intensity
1	3853.08	101.807	2	3838.61	102.003	3	3734.48	101.227
4	3689.16	101.712	5	3675.66	101.808	6	3647.7	101.338
7	3396.99	96.3633	8	2930.31	98.6866	9	1652.7	98.6042
10	1540.85	98.885	11	1432.85	97.1881	12	1337.39	97.9174
13	1281.47	97.5544	14	1261.22	97.1049	15	1184.08	98.0793
16	1067.41	90.9796	17	1038.48	94.6633	18	1026.91	94.6331
19	958.448	93.0955	20	915.058	98.1744	21	829.241	96.5509

Figure S34. IR spectrum of **2**.

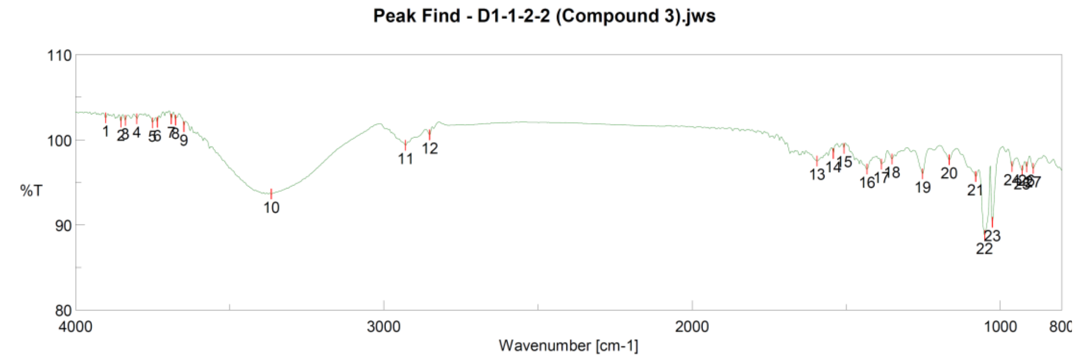

[Result of Peak Picking]

No.	Position	Intensity	No.	Position	Intensity	No.	Position	Intensity
1	3902.25	102.567	2	3853.08	102.065	3	3838.61	102.265
4	3801.01	102.461	5	3750.87	101.993	6	3734.48	102.042
7	3689.16	102.431	8	3675.66	102.32	9	3648.66	101.521
10	3365.17	93.6214	11	2929.34	99.3496	12	2851.24	100.575
13	1594.84	97.489	14	1541.81	98.3765	15	1507.1	98.9793
16	1432.85	96.558	17	1385.6	97.1284	18	1351.86	97.7417
19	1252.54	95.9832	20	1165.76	97.6486	21	1079.94	95.6559
22	1051.01	88.766	23	1025.94	90.2805	24	962.305	96.844
25	929.521	96.3626	26	915.058	96.8062	27	893.844	96.6221

Figure S35. IR spectrum of **3**.

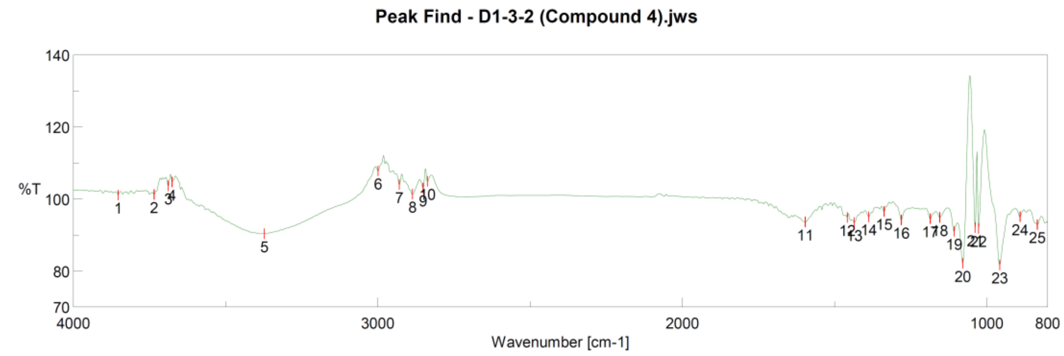

[Result of Peak Picking]

No.	Position	Intensity	No.	Position	Intensity	No.	Position	Intensity
1	3853.08	101.063	2	3734.48	101.201	3	3688.19	103.583
4	3675.66	104.675	5	3372.89	90.3507	6	2998.77	107.751
7	2929.34	103.984	8	2885.95	101.321	9	2851.24	103.04
10	2836.77	104.884	11	1595.81	93.5869	12	1457.92	94.8594
13	1434.78	93.4162	14	1387.53	95.0113	15	1337.39	96.5521
16	1280.5	94.1601	17	1186.01	94.5066	18	1154.19	94.875
19	1107.9	90.9746	20	1078.98	82.1157	21	1038.48	92.0234
22	1026.91	91.7685	23	957.484	81.6238	24	890.952	95.0447
25	834.062	92.8556						

Figure S36. IR spectrum of **4**.

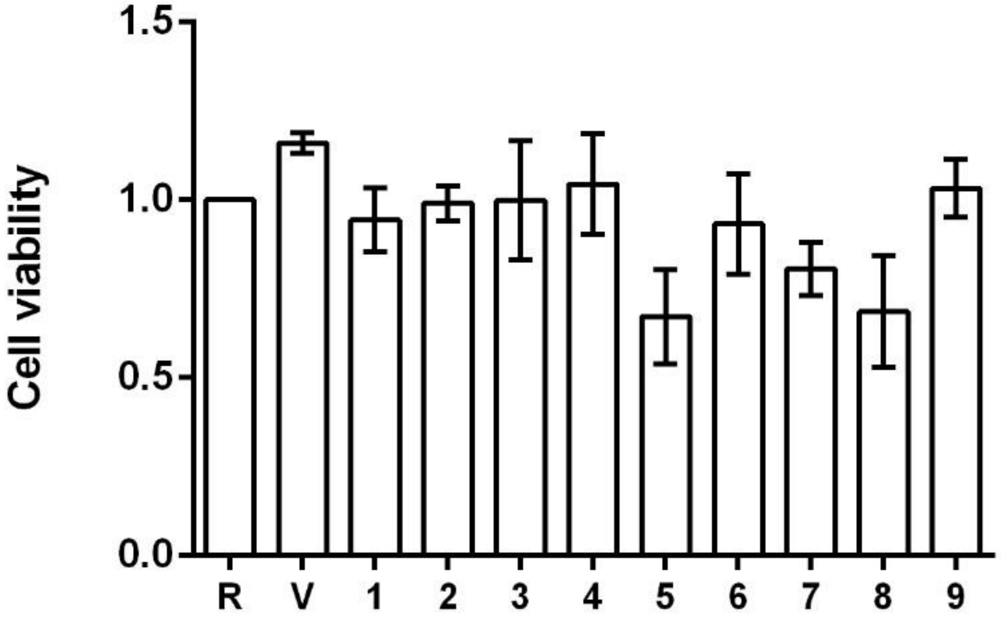

Figure S37. Cell viabillity in human THP-1 monocytic cells.

Physical data of the new compounds

Trichoderminol B (**1**): Colorless oil ; $[\alpha]_D^{25}$ −37.3 (*c* 0.2, MeOH); IR (ν_{max}, KBr): 3365, 2937, 1647, 1457, 1377, 1253, 1045, and 1031 cm^{-1}; ^1H and ^{13}C NMR data: see Table 1; HRESIMS $[M - H_2O + H]^+$ at *m*/*z* 283.1536 (calcd. 283.1540 for $C_{15}H_{23}O_5$).

Trichoderminol C (**2**): Colorless oil; $[\alpha]_D^{25}$ −24.5 (c 0.2, MeOH); IR (ν_{max}, KBr): 3397, 2930, 1652, 1433, 1337, 1261, 1067, and 1038 cm^{-1}; ^1H and ^{13}C NMR data: see Table 1; HRESIMS $[M + H]^+$ at *m*/*z* 267.1588 (calcd. 267.1590 for $C_{15}H_{23}O_4$).

Trichoderminol D (**3**): Colorless gum; $[\alpha]_D^{25}$ −18.3 (*c* 0.2, MeOH); IR (ν_{max}, KBr): 3365, 2929, 2851, 1595, 1433, 1386, 1352, 1252, 1166, 1080, 1051, and 1026 cm^{-1}; ^1H and ^{13}C NMR data: see Table 1; HRESIMS $[M - H_2O + H]^+$ at *m*/*z* 283.1537 (calcd. 283.1540 for $C_{15}H_{23}O_5$).

Trichoderminol E (**4**): Colorless gum; $[\alpha]_D^{25}$ −10.0 (*c* 0.2, MeOH); IR (ν_{max}, KBr): 3372, 2929, 2886, 1596, 1458, 1388, 1281, 1186, 1154, 1107, and 1079 cm^{-1}; ^1H and ^{13}C NMR data: see Table 1; HRESIMS $[M + H]^+$ at *m*/*z* 269.1743 (calcd. 269.1747 for $C_{15}H_{25}O_4$).
